# Effects of exogenous microbial fertilizer and *in situ* green manure management on soil microbial community structure and predicted functional potential in Korla fragrant pear orchards

**DOI:** 10.3389/fmicb.2026.1941612

**Published:** 2026-10-05

**Authors:** Yixin Ke, Pengxue Jia, Xintong Gao, Enduo Liu, Kailong Wang, Zhongping Chai

**Affiliations:** 1 College of Resources and Environment, Xinjiang Agricultural University, Urumqi, China; 2 Xinjiang Key Laboratory of Soil and Plant Ecological Processes, Xinjiang Agricultural University, Urumqi, China

**Keywords:** biofertilizer, green manure, metabolic potential, microbial community, planting density

## Abstract

Long-term chemical fertilizer overuse has degraded soil quality in Korla fragrant pear orchards of Xinjiang, causing reduced organic matter accumulation, nutrient imbalance, and microbial community shifts. However, the effects of exogenous microbial fertilizers and endogenous green manure, particularly under different planting densities, on soil microbiome regulation in sandy orchards of southern Xinjiang remain unclear. Here, a 7–8-year-old Korla fragrant pear orchard was subjected to six fertilization treatments: conventional fertilization (CK), microbial fertilizer (JF), low- and high-density *Melilotus officinalis* (CM1 and CM2), and low- and high-density sunflower (*Helianthus annuus*) (DK1 and DK2). Soil physicochemical properties, 16S rRNA and ITS amplicon sequencing, and PICRUSt2-based functional prediction were integrated to evaluate treatment effects on soil properties, microbial communities, and predicted functional potential. Organic fertilization generally reduced electrical conductivity (EC) and improved organic matter and available nutrients. Among treatments, CM1 showed relatively favorable soil improvement, reducing EC by 19.20% and increasing alkali-hydrolyzable nitrogen and total nitrogen by 36.20 and 36.83%, respectively. JF significantly increased fungal diversity, with the Chao1 index rising by 48.6%, while green manure species and planting density strongly influenced bacterial and fungal community structures. All organic fertilization treatments reduced the relative abundance of *Fusarium*, with CM1 showing the greatest decrease (29.8–60.0%). Functional prediction revealed distinct microbial responses: JF enhanced pathways related to fatty acid β-oxidation and lysine fermentation, with associated gene abundances increasing by 11.86 and 203.32%, respectively; CM1 increased butyrate fermentation-related predicted functions and reduced several denitrification-related predicted functions; DK1 mainly enhanced glycolysis and the tricarboxylic acid cycle, whereas DK2 showed greater potential in nitrogen cycling and phosphorus transformation-related functions. RDA indicated that bacterial functional profiles were associated with soil available potassium, alkali-hydrolyzable nitrogen, and total nitrogen. Under the conditions of this experiment, different fertilization strategies altered soil microbial communities by modifying environmental conditions and microbial assembly patterns. CM1 showed relatively strong potential for improving soil properties and regulating microbial communities, while microbial fertilizer and sunflower treatments exhibited distinct functional responses. These findings provide insights into green manure and microbial fertilizer management in arid-region pear orchards of southern Xinjiang.

## Graphical abstract

CK: Conventional chemical fertilizer (control), JF: Microbial organic fertilizer treatment, DK1: Low-density sunflower (Helianthus annuus), DK2: High-density sunflower (Helianthus annuus), CM1: Low-density Melilotus officinalis, CM2: Highdensity Melilotus officinalis, EC :Electrical Conductivity, SOM :Soil Organic Matter, AK :Available Potassium, AN :Alkaline Hydrolysis Nitrogen, and TN :Total Nitrogen.
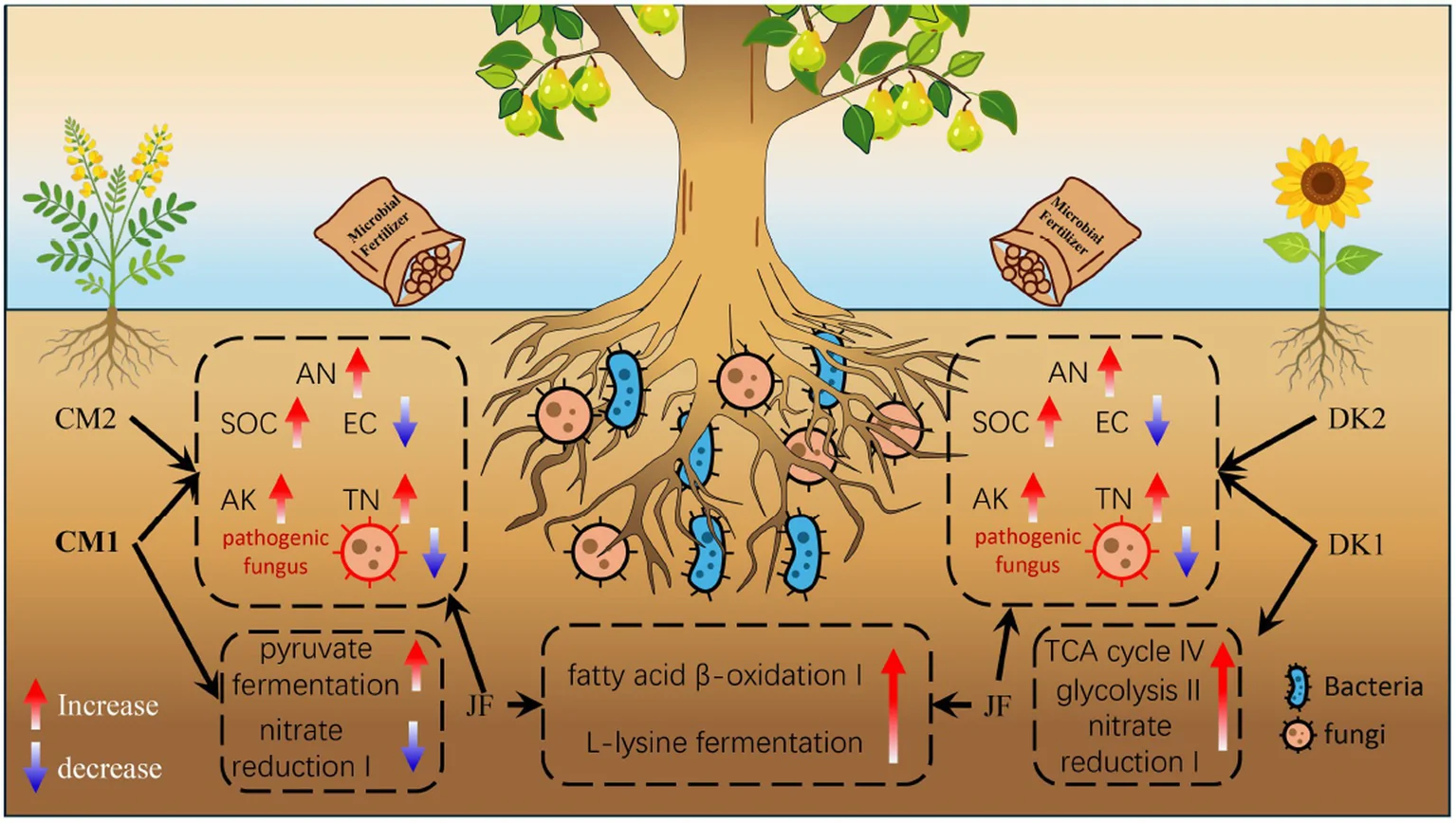

## 1 Introduction

Soil microorganisms constitute the most biologically dynamic component of terrestrial ecosystems, fulfilling indispensable functions in organic matter decomposition, nutrient cycling, soil structural maintenance, and plant health regulation (Iqbal et al., 2025). Bacteria and fungi, as core constituents of the soil microbiota, exert direct and indirect influences on soil fertility and crop growth through a suite of ecological services—including organic matter mineralization, nitrogen fixation, phosphorus and potassium mobilization, and pathogen suppression (Adomako et al., 2022).

As a major modern agricultural base in China, Xinjiang has experienced pronounced soil compaction, progressive salinization, and declining organic matter content in certain regions as a consequence of long-term intensive chemical fertilization. These adverse trends urgently demand the restoration of soil microecological health through the adoption of green fertilization practices (Shi et al., 2023; Abudurezike et al., 2025). Within this context, biofertilizer and green manure—two environmentally benign fertilization technologies—have attracted considerable scholarly and practical interest as pivotal instruments for promoting green and sustainable agricultural development (Yang et al., 2019; Khan et al., 2020).

Korla fragrant pear (*Pyrus sinkiangensis* Yu) is an elite pear cultivar endemic to Xinjiang and holds national recognition as a Geographical Indication product. It occupies a central position in the local specialty fruit industry and serves as a cornerstone sector for enhancing farmers’ incomes and advancing rural revitalization across southern Xinjiang (Zhang et al., 2022). The pear cultivation area in Korla City currently extends to approximately 26,800 hectares (402,000 mu), with an annual output exceeding 370,000 tons and a full-industry-chain value surpassing 6.5 billion RMB. These products are exported to 34 countries and regions along the Belt and Road Initiative (National Bureau of Statistics of China, 2025).

On the global stage, China ranks as the world’s largest producer and exporter of fresh pears, with export volume reaching 707,000 tons in 2025—valued at 630 million USD and accounting for over 30% of global trade volume, thereby consistently leading the international pear market (USDA, 2025).

Nevertheless, intensive cultivation practices persisting over the long term, compounded by excessive reliance on chemical fertilizers, have induced a cascade of soil constraints in pear orchards: structural compaction, aggravated secondary salinization, depletion of organic matter, and disruption of microbial community equilibrium. These degradative processes have, in turn, precipitated declining soil nutrient availability and deterioration of fruit flavor and quality—severely undermining the green and sustainable development of the fragrant pear industry (Abudurezike et al., 2025; Li W. et al., 2025).

Microbial fertilizers and green manures have emerged as promising bio-based approaches for improving soil quality and promoting sustainable agricultural production. Unlike conventional nutrient inputs that mainly provide external resources, microbial fertilizers introduce beneficial microorganisms, such as *Bacillus subtilis*, into the soil ecosystem, allowing them to actively participate in nutrient cycling, synthesize growth-promoting substances, suppress soil-borne pathogens, and ultimately support crop growth and yield formation (Chaudhary et al., 2022; Ahsan et al., 2024; Li J. et al., 2025), while simultaneously improving soil organic matter content, available nutrient pools, and pH conditions (Schenck zu Schweinsberg-Mickan and Müller, 2009; Dębska et al., 2016). The judicious application of microbial fertilizers has been substantiated as an effective strategy for optimizing soil microbial community structure, bolstering crop disease resistance, and ameliorating soil physicochemical properties (Plocek et al., 2024).

In parallel, green manure achieves *in situ* endogenous organic matter input through plant residues and root exudates. The incorporation of green manure crops introduces fresh organic materials into the soil; their root exudates and decomposing residues supply abundant carbon sources and energy for soil microorganisms, thereby elevating microbial biomass and metabolic activity while driving the restructuring of community composition (Zhao et al., 2012; Muhammad et al., 2021; Thapa et al., 2021). Nonetheless, the nutrient input pathways of exogenous biofertilizer and endogenous green manure are fundamentally distinct, and the differential mechanisms through which these two amendment types regulate soil microbial communities remain largely unresolved.

A substantial body of research has explored the roles of microbial fertilizers and green manures in improving soil quality and crop performance across various horticultural systems, including apple (Wang et al., 2020; Ding et al., 2021), citrus (Qiu et al., 2021; Castellano-Hinojosa et al., 2023), strawberry (Li et al., 2022; Shrestha et al., 2024), and peach orchards (Ramos et al., 2022; Li Z. et al., 2025). However, research on Korla fragrant pear orchards has not yet kept pace with these advances. Existing studies have primarily examined how different fertilization practices affect soil bacterial dynamics and orchard productivity, while the broader microbial responses following green manure incorporation remain unclear. In particular, how bacterial and fungal communities jointly respond to green manure application, and whether green manure species and planting density can reshape microbial functional potential, are questions that have received limited attention.

Previous investigations of green manure utilization in Korla fragrant pear orchards have largely concentrated on visible outcomes, such as fruit quality improvement (Han et al., 2022; Ke et al., 2025), soil enzyme activity changes (He et al., 2025), and yield-related traits. Yet, the microbial processes occurring beneath the soil surface—where nutrient cycling and ecological functions are continuously regulated—remain insufficiently understood. This gap becomes more evident when considering that most green manure experiments have adopted a single planting density, leaving the effects of density optimization largely unexplored. For example, do different sowing densities of *Melilotus officinalis* and sunflower trigger distinct microbial assembly patterns in the sandy soils of southern Xinjiang? Under the unique conditions of arid-region pear orchards, the comparative effects of exogenous microbial fertilizers and green manures with different species and densities on soil microbial composition and predicted functional potential have yet to be systematically clarified.

To fill these knowledge gaps, this study selected a 7–8-year-old Korla fragrant pear orchard as the research platform and established six fertilization treatments: conventional chemical fertilization (CK), microbial fertilizer (JF), low-density and high-density *M. officinalis* (CM1 and CM2), and low-density and high-density sunflower (*Helianthus annuus*) (DK1 and DK2). We combined soil physicochemical analyses, including measurements of soil organic matter, total nitrogen, available nutrients, and electrical conductivity, with high-throughput sequencing of 16S rRNA and ITS amplicons to characterize bacterial and fungal community composition, α/β diversity, and key microbial biomarkers. In addition, PICRUSt2 functional prediction was applied to explore potential microbial metabolic pathways and functional shifts under different fertilization regimes.

Specifically, this study aimed to (1) clarify how exogenous microbial fertilizer and green manures with different species and planting densities influence soil physicochemical properties in sandy Korla fragrant pear orchards; (2) compare the responses of bacterial and fungal communities, as well as dominant microbial taxa, to microbial fertilizer and green manure applications; (3) reveal the changes in microbial community structure and predicted functional potential driven by green manure type and planting density; and (4) evaluate the relative advantages of different fertilization strategies in improving soil quality and regulating microbial communities. The findings are expected to provide a scientific basis for optimizing green manure selection, refining organic fertilization practices, and promoting sustainable cultivation of pear orchards in the arid region of southern Xinjiang.

## 2 Materials and methods

### 2.1 Experimental site description

The field experiment was conducted in Heshilike Township, Korla City, Bayingolin Mongol Autonomous Prefecture, Xinjiang Uygur Autonomous Region, China (86°11′23.34″E, 41°44′26.04″N; altitude 855.3 m). The experimental site is situated in central Xinjiang, at the southern foot of the Tianshan Mountains and the northeastern edge of the Tarim Basin, bordered to the north by the Tianshan mountain ranges and to the south by the Taklimakan Desert—the world’s second-largest shifting sand desert. The region exhibits a warm temperate continental climate, characterized by substantial diurnal temperature fluctuations and abundant solar radiation, with an average annual sunshine duration of 2,990 h. The mean annual temperature ranges from 14 to 15 °C, accompanied by mean annual precipitation of 50–58 mm and maximum annual evaporation of 2,788.2 mm. The effective accumulated temperature (≥10 °C) spans 4,100–4,400 °C, and the frost-free period extends from 210 to 239 days, with northeasterly winds prevailing throughout the year. The soil texture of the pear orchard is sandy, with baseline physicochemical properties as follows: pH 7.80, organic matter 11.22 g·kg^−1^, available phosphorus 12.06 mg·kg^−1^, available potassium 167.17 mg·kg^−1^, and alkaline hydrolysis nitrogen 16.63 mg·kg^−1^.

### 2.2 Plant material and experimental design

The field experiment was conducted during 2023–2024, employing 7- to 8-year-old Korla fragrant pear trees grafted onto Pyrus betulifolia rootstocks. Trees were planted at a spacing of 3 m × 5 m, corresponding to a density of 675 trees·hm^−2^. Six treatments were established in the pear orchard: conventional chemical fertilizer (CK); biofertilizer containing *Bacillus subtilis* and *Bacillus licheniformis* (JF); sweet clover sown at a row spacing of 25 cm with a seeding rate of 21 kg·hm^−2^ (CM1); sweet clover sown at a row spacing of 20 cm with a seeding rate of 27 kg·hm^−2^ (CM2); oil sunflower sown at a row spacing of 25 cm with a seeding rate of 27 kg·hm^−2^ (DK1); and oil sunflower sown at a row spacing of 20 cm with a seeding rate of 33 kg·hm^−2^ (DK2). Fertilizer application rates and detailed green manure species and sowing specifications are presented in Table 1, and the experimental layout is illustrated in Figure 1.

**Table 1:** Experimental treatment design plan.

Treatment	Green manure type	Seeding rate (kg/hm^2^)	Sowing depth (cm)	Row spacing (cm)	Agricultural microbial fertilizer (kg/hm^2^)	Nutrient application rate kg/hm^2^		
						*N*	P_2_O_5_	K_2_O
CK	None	0	0	0	0	300	300	150
JF	None	0	0	0	1,200	300	300	150
DK1	Oil Sunflower	27	2–3	25	0	300	300	150
DK2	Oil Sunflower	33	2–3	20	0	300	300	150
CM1	Sweetclover	21	1	25	0	300	300	150
CM2	Sweetclover	27	1	20	0	300	300	150

**Figure 1:**
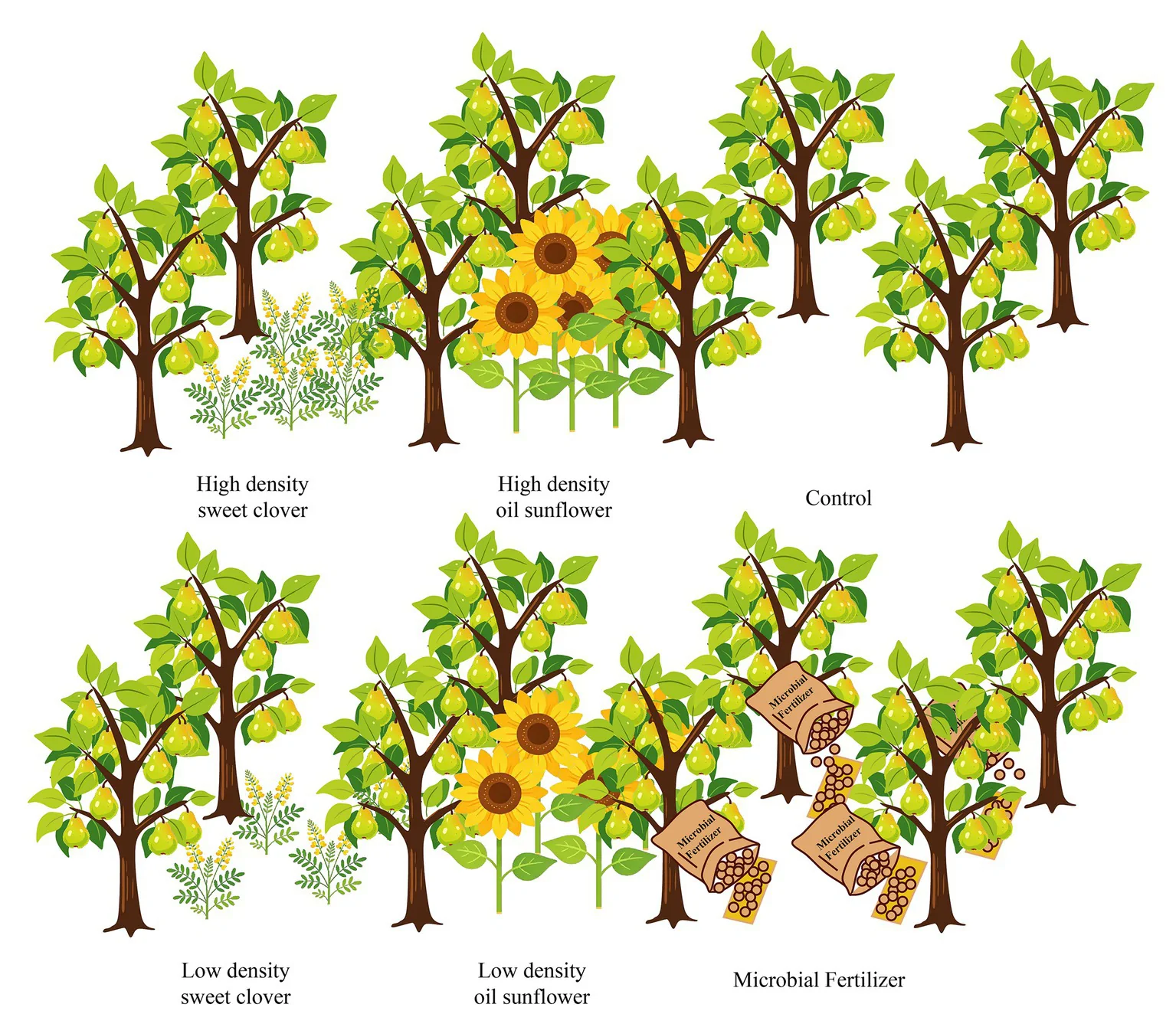
Schematic diagram of Korla fragrant pear planting configuration and organic fertilization treatments.

Each treatment consisted of three independent field plots as biological replicates. Each experimental treatment covered an area of 666.67 m^2^, and the total experimental area was 12,000 m^2^ (Figure 2). Sheep manure at a rate of 15,000 kg·hm^−2^ was applied as a one-time basal application in autumn. Phosphorus and potassium fertilizers were applied as single superphosphate (containing 46% P₂O₅) and potassium sulfate (containing 51% K₂O), respectively, both of which were incorporated as a single basal dressing prior to spring bud break. Nitrogen fertilizer was applied as urea (containing 46% N). The biofertilizer employed was Shipulang (produced by Zhushang Fertilizer Co., Ltd., Qingdao, China), which is enriched with *Bacillus subtilis* and *Bacillus licheniformis* at a viable cell count ≥ 5.0 × 10^8^ CFU·g^−1^. For both nitrogen fertilizer and biofertilizer, 60% of the total amount was applied before bud break, with the remaining 40% top-dressed during the early fruit expansion stage. Fertilizers were applied in ring-shaped furrows approximately 30 cm in both depth and width, dug at a distance of 50–80 cm from the central trunk, and evenly distributed within the furrows.

**Figure 2:**
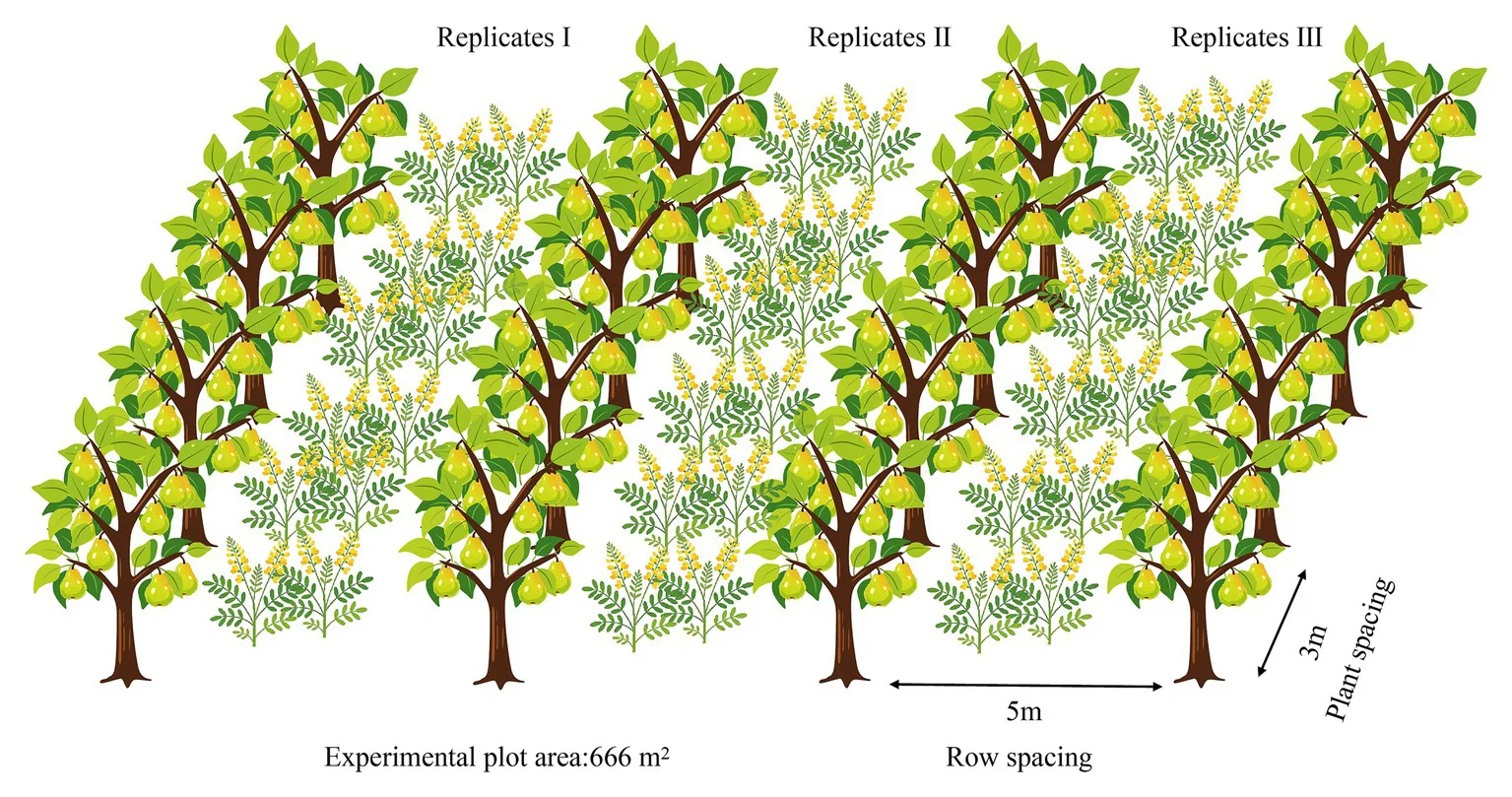
Experimental plot design. This figure illustrates the layout of the experimental plots. The *Melilotus officinalis* plants shown in the figure are for schematic representation only; the green manure species and planting densities varied among different plots.

The green manure species cultivated were sweet clover (*Melilotus officinalis*) and oil sunflower (*Helianthus annuus*), with seeds sourced from the Inner Mongolia Autonomous Region and purchased from Gansu Lanbin Ecological Technology Co., Ltd. Seed purity and germination rates were 95 and 85% for sweet clover, and 90 and 80% for oil sunflower, respectively. A spacing of 70–80 cm was maintained between pear tree rows and green manure strips. Green manure seeds were sown in early April, and the aboveground biomass was mechanically shredded and incorporated into the soil by ploughing in late July.

### 2.3 Sample collection and processing

Soil samples were collected from the pear orchard at 100 days after green manure incorporation. Following the removal of surface litter, samples were collected from the 0–20 cm layer on both sides of the fertilization furrows. The samples from the same depth on both sides of each furrow were pooled into a single composite sample. After preliminary crushing and homogenization, the samples were placed in sealed bags, transferred to insulated coolers containing dry ice, and transported to the laboratory under refrigeration. Upon arrival, the samples were passed through a 2 mm sieve after removing plant roots and coarse stones, thoroughly mixed, and stored at −80 °C. Within one week, aliquots were delivered to Shanghai Personal Biotechnology Co., Ltd. for microbial community structure analysis. The remaining portion of each sample was air-dried indoors and passed through a 1 mm sieve for soil physicochemical analysis.

### 2.4 Analytical methods

#### 2.4.1 Soil physicochemical property determination

Soil physicochemical parameters were measured following the methods described by Bao (2000) in Soil and Agricultural Chemistry Analysis. Electrical conductivity (EC) was determined using a conductivity meter. Total nitrogen (TN), soil organic matter (SOM), alkaline hydrolysis nitrogen (AN), and available potassium (AK) were determined by the semi-micro Kjeldahl method, potassium dichromate external heating method, alkaline diffusion method, and ammonium acetate extraction–flame photometry method, respectively.

#### 2.4.2 Sequencing and bioinformatic analysis of soil bacterial and fungal communities

DNA extraction and PCR amplification: Total genomic DNA was extracted from soil samples using the OMEGA Soil DNA Kit (M5635-02) (OMEGA Bio-Tek, Norcross, GA, USA) following the manufacturer’s instructions and stored at −20 °C prior to further analysis. DNA quantity and quality were assessed using a NanoDrop NC2000 spectrophotometer (Thermo Fisher Scientific, Waltham, MA, USA) and agarose gel electrophoresis, respectively. For fungi, the ITS1 region of the ITS gene was amplified using the forward primer ITS1F (5′-GGAAGTAAAAGTCGTAACAAGG-3′) and reverse primer ITS1R (5′-GCTGCGTTCTTCATCGATGC-3′). For bacteria, the V3–V4 region of the 16S rRNA gene was amplified using the forward primer 338F (5′-ACTCCTACGGGAGGCAGCA-3′) and reverse primer 806R (5′-GGACTACHVGGGTWTCTAAT-3′). Multiplex sequencing was achieved by incorporating sample-specific barcodes into the primers. PCR reactions were conducted using NEB Q5 high-fidelity DNA polymerase. The thermal cycling protocol comprised initial denaturation at 98 °C for 30 s, followed by 28 cycles of denaturation at 98 °C for 15 s, annealing at 50 °C for 30 s, and extension at 72 °C for 30 s, with a final extension at 72 °C for 5 min and subsequent holding at 4 °C. PCR products were subjected to 2% agarose gel electrophoresis, and target fragments were excised and purified using the Axygen gel extraction kit.

High-throughput sequencing analysis: PCR products were quantified using the Quant-iT PicoGreen dsDNA Assay Kit on a Microplate Reader (BioTek, FLx800) and pooled in equimolar ratios according to the required sequencing data volume per sample. Libraries were constructed using the Illumina TruSeq Nano DNA LT Library Prep Kit. Following quality inspection and quantification, qualified libraries were subjected to 2 × 250 bp paired-end sequencing on the MiSeq platform using the MiSeq Reagent Kit V3 (600 cycles). Microbiome bioinformatics analyses were performed using QIIME2 (version 2019.4). Briefly, raw sequence data were demultiplexed using the demux plugin, followed by primer removal using the cutadapt plugin. The DADA2 plugin was subsequently applied for quality filtering, model-based denoising, paired-end read merging, and chimera removal. Unlike conventional sequence clustering approaches, DADA2 inferred exact amplicon sequence variants (ASVs) based on an error correction model. Representative ASV sequences and corresponding abundance tables were generated for downstream taxonomic classification and microbial community analyses. Taxonomic assignment of ASVs was accomplished by comparing representative sequences against the UNITE 9 database^1^ for fungi and the Silva 138.1 database^2^ for bacteria. ASVs with relative abundances below 0.001% of the total sequencing depth across all samples were excluded, and the resulting ASV abundance matrix was employed for subsequent analyses. Taxonomic composition bar plots were generated using R software to visualize the number of ASVs and taxonomic identification results across samples. All sequencing and initial bioinformatics analyses were performed by Shanghai Personal Biotechnology Co., Ltd.

Functional prediction: Functional prediction: Functional potential prediction was performed based on amplicon sequencing data. For bacterial communities, PICRUSt2 (v2.5.2) was applied to 16S rRNA gene sequencing data to predict functional profiles. KEGG Orthologs (KO) were used as functional identifiers, and the predicted functions were further mapped to the MetaCyc database to obtain pathway abundance matrices for each sample. For fungal communities, ITS ASV representative sequences and corresponding abundance tables were used as input datasets for functional potential prediction. The predicted functional profiles were further annotated against the MetaCyc database to obtain predicted metabolic pathway abundance matrices. The predicted pathway abundance data were normalized and subjected to statistical analyses among different treatments. These predicted functional profiles represent potential functional characteristics inferred from amplicon sequencing data rather than directly measured gene expression or metabolic activity.

### 2.5 Data processing and statistical analysis

The field experiment was conducted continuously from 2023 to 2024, with fertilization treatments established in the first year and soil samples collected in the second year (2024) after green manure incorporation. All treatments were conducted with three independent biological replicates corresponding to three field plots. Data are presented as means ± standard errors. Statistical analyses and visualization were performed using Excel 2019, IBM SPSS Statistics 27, R version 4.3.2, and the Genescloud platform.^3^ Differences among treatments were evaluated using appropriate statistical methods according to data distribution characteristics, and detailed statistical procedures are described in the Supplementary methods. Microbial community analyses were performed based on ASV abundance data. Community composition differences among treatments were evaluated using Bray–Curtis distance-based non-metric multidimensional scaling (NMDS) and permutational multivariate analysis of variance (PERMANOVA) implemented with the vegan package in R (Oksanen et al., 2026). Venn diagrams were generated using the VennDiagram package (Chen and Boutros, 2011), heatmaps were constructed using the pheatmap package (Kolde, 2025), and redundancy analysis (RDA) was performed using the vegan package (Oksanen et al., 2026). Differential abundance analysis of bacterial and fungal taxa was conducted using ANCOM-BC2 based on raw ASV count data to account for compositional bias and sequencing depth variation in amplicon sequencing datasets. Multiple testing correction was performed using the Benjamini–Hochberg method, with q < 0.05 considered statistically significant. LEfSe analysis (Segata et al., 2011) was performed on the Genescloud platform as an exploratory approach to identify potential microbial biomarkers among different treatments. Taxa with *p* < 0.05 and LDA score > 3 were considered discriminative features, and the robustness of LEfSe-identified taxa was further evaluated by comparison with ANCOM-BC2 results. Potential bacterial functional profiles were predicted using PICRUSt2, while fungal ecological guilds were inferred using FUNGuild. Functional prediction results were interpreted as potential ecological characteristics rather than direct evidence of microbial activity.

## 3 Results and analysis

### 3.1 Soil physicochemical properties

Different fertilizer sources and green manure planting densities significantly reshaped the physicochemical characteristics of soil in the Korla fragrant pear orchard (Supplementary Figure S1). Both microbial fertilizer and green manure applications contributed to soil nutrient improvement, but the magnitude and direction of these effects varied among treatments. Compared with microbial fertilizer, green manure generally showed a stronger capacity to enhance soil fertility. Meanwhile, different green manure species did not respond identically to changes in planting density, indicating that the influence of density was closely linked to plant species characteristics.

Soil electrical conductivity (EC) varied markedly among the different treatments. Except for DK2, all fertilization treatments reduced EC compared with CK, with decreases of 8.58, 19.20, 7.38, and 8.00% observed in JF, CM1, CM2, and DK1, respectively. Among all treatments, CM1 produced the most pronounced reduction, lowering EC by 19.20% relative to CK and reaching a significantly lower level than the other treatments (*p* < 0.05). In contrast, DK2 maintained the highest EC value, showing no significant difference from CK. Interestingly, increasing green manure density did not lead to a uniform response pattern. For *Melilotus officinalis*, EC increased from 1125.00 μS·cm^−1^ in CM1 to 1289.67 μS·cm^−1^ in CM2, whereas sunflower treatments only showed an upward tendency from DK1 to DK2. These results suggest that the effect of planting density on soil salt accumulation or migration depends strongly on green manure species.

Soil organic matter (SOM) content increased significantly under all fertilization treatments compared with CK (*p* < 0.05). JF, CM1, CM2, DK1, and DK2 increased SOM by 16.76, 25.91, 21.75, 33.15, and 26.32%, respectively. DK1 achieved the greatest improvement, with SOM increasing by 33.15%, whereas JF showed the smallest increase. Overall, green manure treatments promoted greater organic matter accumulation than microbial fertilizer, highlighting the contribution of plant-derived biomass inputs to soil carbon enrichment. When comparing different planting densities, CM2 showed a slight decline in SOM compared with CM1, although the difference was not significant. In contrast, increasing sunflower density from DK1 to DK2 significantly reduced SOM from 14.87 to 14.11 g·kg^−1^, suggesting that organic matter accumulation under sunflower cultivation was more sensitive to density changes.

The incorporation of green manure generally promoted soil available potassium (AK) accumulation. Except for DK2, all fertilization treatments significantly increased AK content compared with CK (*p* < 0.05), with increases of 11.23, 20.04, 14.72, and 18.40% recorded in JF, CM1, CM2, and DK1, respectively. CM1 and DK1 exhibited relatively stronger enhancement effects, whereas DK2 decreased AK content to 159.00 mg·kg^−1^, even lower than that in CK. However, no significant differences were detected between different planting densities of the same green manure species, indicating that density variation exerted only a limited influence on soil available potassium.

Alkali-hydrolyzable nitrogen (AN) showed a more sensitive response to both fertilizer type and green manure density. All fertilization treatments significantly increased AN compared with CK, with increases of 8.84, 36.14, 23.29, 22.79, and 16.29% observed in JF, CM1, CM2, DK1, and DK2, respectively. CM1 exhibited the highest AN content and significantly exceeded all other treatments, whereas JF showed the weakest improvement. Increasing green manure density did not further enhance nitrogen accumulation; instead, AN decreased by 9.48% in CM2 compared with CM1 and by 5.31% in DK2 compared with DK1. This pattern suggests that simply increasing biomass input does not necessarily translate into greater soil nitrogen availability.

Total nitrogen (TN) was mainly determined by fertilizer source rather than planting density. All fertilization treatments significantly increased TN compared with CK, with JF, CM1, CM2, DK1, and DK2 increasing TN by 9.09, 36.81, 32.55, 29.21, and 29.23%, respectively. Although no significant differences were observed among the four green manure treatments, all green manure applications resulted in higher TN contents than microbial fertilizer. This indicates that green manure incorporation provided a more effective pathway for enhancing soil nitrogen storage, while increasing planting density alone was insufficient to further alter TN levels.

Taken together, endogenous green manure exhibited stronger soil fertility improvement potential than exogenous microbial fertilizer under the conditions of this study. However, increasing green manure density did not always generate additional benefits, and its effects depended largely on species identity. High-density *M. officinalis* primarily increased soil EC and reduced alkali-hydrolyzable nitrogen, whereas high-density sunflower mainly resulted in decreases in SOM and alkali-hydrolyzable nitrogen. These findings indicate that green manure species determine how soil nutrient dynamics respond to planting density adjustments, emphasizing the need to consider both plant type and density when designing sustainable fertilization strategies for arid-region orchards.

### 3.2 Soil microbial ASV abundance

In this study, the rarefaction curves for both bacterial and fungal communities across all treatments exhibited a steep initial rise, followed by progressive deceleration and eventual plateauing (Figure 3). The bacterial rarefaction curves reached asymptote once sequencing depth exceeded 20,000 reads, beyond which no appreciable increase in observed species was detected (Figure 3a). Likewise, the fungal rarefaction curves approached saturation at approximately 40,000 reads, indicating that species detection had attained near-complete coverage (Figure 3b).

**Figure 3:**
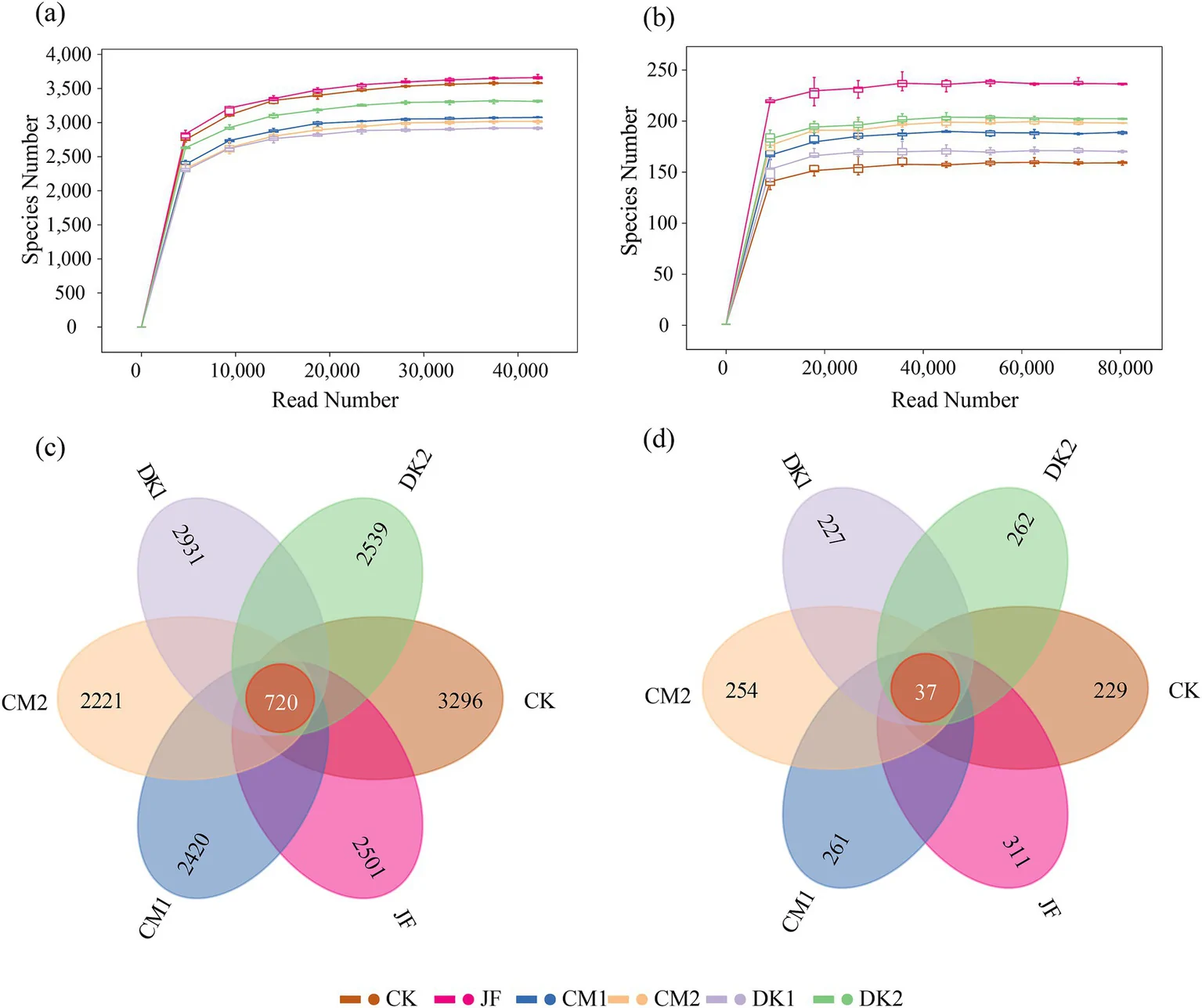
Rarefaction curves showing the number of observed taxa **(a,b)** and Venn diagrams illustrating the distribution of unique and shared ASVs **(c,d)** for bacterial **(a,c)** and fungal **(b,d)** communities across different fertilization treatments.

Across the six treatments, a total of 15,908 bacterial ASVs and 1,544 fungal ASVs were identified (Figures 3c,d). A total of 720 bacterial ASVs (4.5% of the total) were shared among all treatments, whereas the number of unique bacterial ASVs varied considerably across treatments. Among these, CK harbored the highest number of unique bacterial ASVs (3,296), followed by DK1 (2,931), DK2 (2,539), JF (2,501), CM1 (2,420), and CM2 (2,221). For fungal communities, 37 ASVs were common to all treatments, representing 2.3% of the total. JF contained the greatest number of unique fungal ASVs (311), followed in descending order by DK2 (262), CM1 (261), CM2 (254), CK (229), and DK1 (227).

### 3.3 Soil microbial α-diversity

Six core indices—Chao1, Goods_coverage, Observed_species, Pielou_e, Shannon, and Simpson—were employed to systematically characterize the effects of different treatments on soil microbial community α-diversity, with the results summarized in Table 2. The Goods_coverage indices for both bacteria and fungi were ≥ 0.99 across all samples, confirming that the sequencing depth was sufficient to adequately capture the true microbial community diversity.

**Table 2:** Alpha diversity of soil bacterial (a) and fungal (b) communities under different fertilization treatments.

	Treatment	Goods_coverage	Chao1	Observed_species	Pielou_e	Shannon	Simpson
a	CK	0.99 ± 0 a	3580.1 ± 121.69 a	3378.7 ± 106.47 a	0.9 ± 0 a	10.57 ± 0.09 ab	0.999 ± 0 a
	JF	0.99 ± 0 a	3665.15 ± 29.81 a	3448.9 ± 23.41 a	0.9 ± 0 a	10.61 ± 0.02 a	0.998 ± 0 a
	CM1	0.99 ± 0 a	3077.21 ± 121 bc	2953.33 ± 96.37 bc	0.89 ± 0 ab	10.3 ± 0.03 bc	0.998 ± 0 a
	CM2	0.99 ± 0 a	3015.92 ± 146.46 bc	2861.37 ± 130.05 bc	0.88 ± 0.01 b	10.12 ± 0.1 c	0.997 ± 0.001 a
	DK1	0.99 ± 0 a	2919.13 ± 173.52 c	2807.5 ± 154.71 c	0.89 ± 0 ab	10.22 ± 0.1 c	0.998 ± 0 a
	DK2	0.99 ± 0 a	3313.1 ± 95.7 ab	3172.63 ± 83.71 ab	0.91 ± 0.01 a	10.54 ± 0.12 ab	0.999 ± 0 a
b	CK	0.99 ± 0 a	159.08 ± 12.87 d	157.13 ± 13.09 d	0.66 ± 0 a	4.81 ± 0.07 c	0.93 ± 0 a
	JF	0.99 ± 0 a	236.32 ± 6.22 a	235.2 ± 6.31 a	0.69 ± 0.01 a	5.46 ± 0.11 a	0.95 ± 0 a
	CM1	0.99 ± 0 a	188.92 ± 14 bcd	186.8 ± 14.03 bcd	0.65 ± 0.01 a	4.9 ± 0.05 bc	0.93 ± 0 a
	CM2	0.99 ± 0 a	197.96 ± 11.99 bc	196.83 ± 12.08 bc	0.66 ± 0.04 a	5.06 ± 0.31 abc	0.93 ± 0.03 a
	DK1	0.99 ± 0 a	170.41 ± 3.66 cd	168.77 ± 3.83 cd	0.68 ± 0.02 a	5 ± 0.14 abc	0.94 ± 0.01 a
	DK2	0.99 ± 0 a	202.12 ± 5.48 b	201.23 ± 5.11 b	0.70 ± 0 a	5.36 ± 0.05 ab	0.95 ± 0 a

Data are presented as mean ± standard error (*n* = 3), with a significance level of *p* = 0.05. Different lowercase letters in the same column indicate significant differences (*p* < 0.05).

Regarding species richness, the bacterial Chao1 and Observed_species indices of JF showed no significant differences from those of CK, yet both were significantly higher than those of CM1, CM2, and DK1 (*p* < 0.05). The corresponding indices of DK2 were significantly greater than those of CM2 and DK1 (*p* < 0.05), but did not differ significantly from CK or JF. Conversely, DK1 exhibited significantly lower Chao1 and Observed_species indices than CK, JF, and DK2 (*p* < 0.05). Collectively, the overall ranking of bacterial species richness was JF > CK > DK2 > CM1 > CM2 > DK1.

For fungal communities, the Chao1 and Observed_species indices exhibited identical trends across treatments. JF displayed significantly higher values than all other treatments (*p* < 0.05), and DK2 showed significantly higher values than CK, CM1, and DK1 (*p* < 0.05). Notably, CK recorded the lowest Chao1 and Observed_species indices among all treatments (*p* < 0.05). The overall ranking of fungal species richness was JF > DK2 > CM2 > CM1 > DK1 > CK.

With respect to species evenness and diversity, the bacterial Pielou_e index of CM2 was significantly lower than that of CK, JF, and DK2, whereas CM1 and DK1 showed no significant differences from the other treatments. In contrast, no significant differences in fungal Pielou_e indices were detected among any of the treatments. The bacterial Shannon index of JF was the highest among all treatments, significantly exceeding those of CM1, CM2, and DK1 (*p* < 0.05). CM2 and DK1 exhibited the lowest Shannon indices, significantly lower than CK, JF, and DK2 (*p* < 0.05), yielding the overall ranking JF > CK > DK2 > CM1 > DK1 > CM2. For fungal communities, JF again demonstrated the highest Shannon index, significantly higher than CK and CM1 (*p* < 0.05), but not significantly different from DK2 (*p* > 0.05). CK exhibited the lowest Shannon index, significantly lower than JF and DK2 (*p* < 0.05), with no significant differences observed among the remaining treatments. The overall ranking of fungal Shannon diversity was JF > DK2 > CM2 > DK1 > CM1 > CK. The Simpson indices for both bacteria and fungi showed no significant differences across treatments, ranging from 0.997 to 0.999 across all samples.

### 3.4 Soil microbial β-diversity

NMDS analysis revealed clear differences in microbial community distribution among the fertilization treatments (Figure 4). The stress values of bacterial and fungal communities were 0.0395 and 0.19, respectively. The bacterial NMDS ordination showed an excellent fit, as indicated by the low stress value, whereas the fungal ordination remained within an acceptable interpretation range with a stress value below 0.20. PERMANOVA analysis based on Bray–Curtis distances further confirmed that fertilization treatments significantly reshaped both bacterial and fungal community structures. For bacterial communities, the treatment effect was highly significant (pseudo-*F* = 4.30, R^2^ = 0.64, *p* = 0.001), accounting for 64% of the variation in community composition. Similarly, fungal communities also responded significantly to different treatments (pseudo-*F* = 2.12, R^2^ = 0.47, *p* = 0.001), with treatment differences explaining 47% of the observed variation.

**Figure 4:**
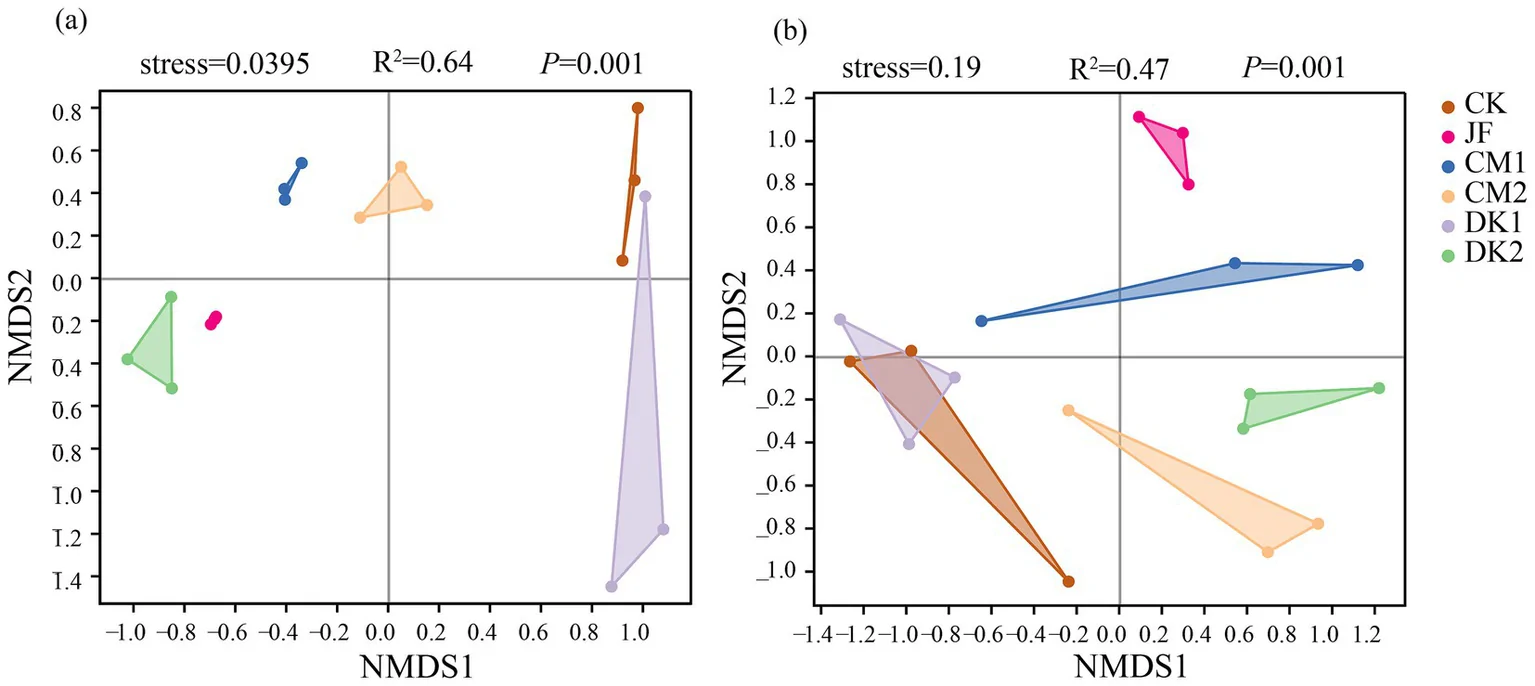
Beta diversity of soil bacterial **(a)** and fungal **(b)** communities under different fertilization treatments (NMDS analysis).

The NMDS ordination plots provided a more intuitive view of these community shifts. Bacterial communities under different treatments showed relatively distinct spatial separation. Samples from CK and DK1 were mainly grouped on the right side of the ordination space, while CM1 and CM2 samples were positioned in the upper-middle region. In contrast, JF and DK2 communities were primarily distributed on the left side of the plot, indicating that these treatments drove bacterial communities toward different compositional patterns. Fungal communities displayed a similar trend, although the separation among treatments was less pronounced. JF samples were mainly located in the upper-right region, CM1 samples clustered in the middle-right area, and DK2 samples were distributed in the lower-right region. Meanwhile, CK and DK1 samples were concentrated on the left side with partial overlap, suggesting relatively similar fungal community structures between these two treatments.

Taken together with the PERMANOVA results, the NMDS ordination demonstrated that different fertilization strategies significantly altered both bacterial and fungal community compositions. However, the magnitude of these effects differed between microbial groups, with fertilization treatments explaining a larger proportion of variation in bacterial communities than in fungal communities. These results indicate that bacterial communities exhibited stronger sensitivity to fertilization-induced environmental changes, whereas fungal communities showed comparatively more stable response patterns.

### 3.5 Relative abundance of dominant soil microbial phyla

At the bacterial level (Figure 5a), the ten most abundant phyla—in descending order of relative abundance—were *Proteobacteria, Gemmatimonadota, Actinobacteriota, Acidobacteriota, Chloroflexi, Bacteroidota, Myxococcota, Patescibacteria, Methylomirabilota*, and *Planctomycetota*. Among these, *Proteobacteria* predominated across all treatments, with relative abundances ranging from 23 to 35%—peaking in DK1 (35%) and reaching their lowest values in JF and DK2 (23%). *Gemmatimonadota* abundance varied from 14 to 25%, attaining its maximum in CM2 (22%)—a 57.1% increase relative to CK (14%). *Actinobacteriota*, with abundances spanning 10 to 20%, was significantly enriched in JF (19%) and DK2 (20%). *Acidobacteriota* exhibited abundances between 8 and 15%, with the minimum recorded in DK1 (8%), whereas Chloroflexi remained comparatively stable, ranging from 9 to 15%.

**Figure 5:**
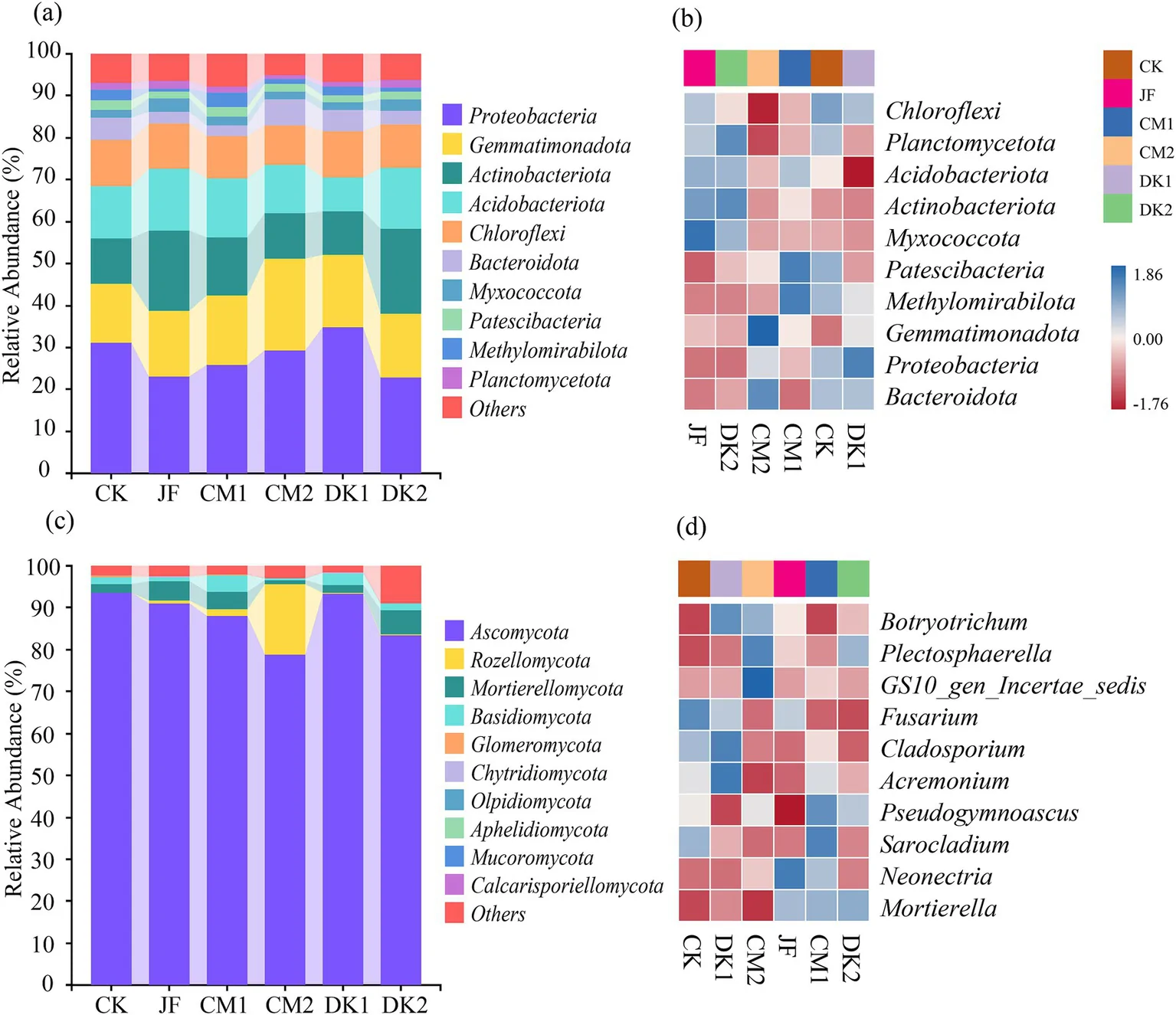
Relative abundance of dominant bacterial **(a)** and fungal **(b)** phyla under different fertilization treatments.

In fungal communities (Figure 5c), the ten most dominant phyla were *Ascomycota, Rozellomycota, Mortierellomycota, Basidiomycota, Glomeromycota, Chytridiomycota, Ophiomycota, Aphelidiomycota, Mucoromycota, and Calcarisporiellomycota. Ascomycota* was overwhelmingly dominant across all treatments, with relative abundances ranging from 78.7 to 93.5%—highest in CK (93.5%) and DK1 (93.3%), yet declining markedly in CM2 to 78.7%, concomitant with a sharp rise in *Rozellomycota* (17.0%).

### 3.6 Relative abundances of dominant bacterial taxa and fungal genera in soil communities

As shown in Figure 6, the bacterial community composition at the taxonomic level (Figure 6a) was dominated by a set of highly abundant taxa. The top 15 dominant bacterial taxa included S0134 terrestrial group, *Vicinamibacteraceae, MND1, MB-A2-108, Sphingomonas, KD4-96, Rokubacteriales, Subgroup_7, Gitt-GS-136, Subgroup_10, Gemmatimonas, AKAU4049, A4b, Gaiella*, and *S085*. Different treatments exerted differential selective effects on the composition of dominant bacterial taxa: JF preferentially enriched *Vicinamibacteraceae, Gitt-GS-136*, and *Gaiella*; CM1 was distinguished by the enrichment of *MND1, Rokubacteriales*, and *S085*; CM2 exhibited elevated relative abundances of *S0134* terrestrial group, *Sphingomonas, Gemmatimonas*, and *AKAU4049*; DK1 yielded exclusive enrichment of *A4b*; whereas DK2 enriched *MB-A2-108, Subgroup_7, Gitt-GS-136*, and *Gemmatimonas*. With respect to fertilizer sources, JF activated specific taxa within *Acidobacteriota* and *Actinobacteriota*, whereas sweet clover (CM1) recruited a greater proportion of saprophytic or symbiotic bacteria. Regarding density effects, the functional genus composition shifted from *MND1/Rokubacteriales* in CM1 to *S0134/Sphingomonas/Gemmatimonas* in CM2, suggesting a transition in microbial life-history strategy from organic matter decomposition toward stable carbon sequestration. For oil sunflower, the transition from DK1 to DK2 transformed a singular enrichment of A4b into a more diverse assemblage comprising MB-A2-108, Subgroup_7, and Gitt-GS-136.

**Figure 6:**
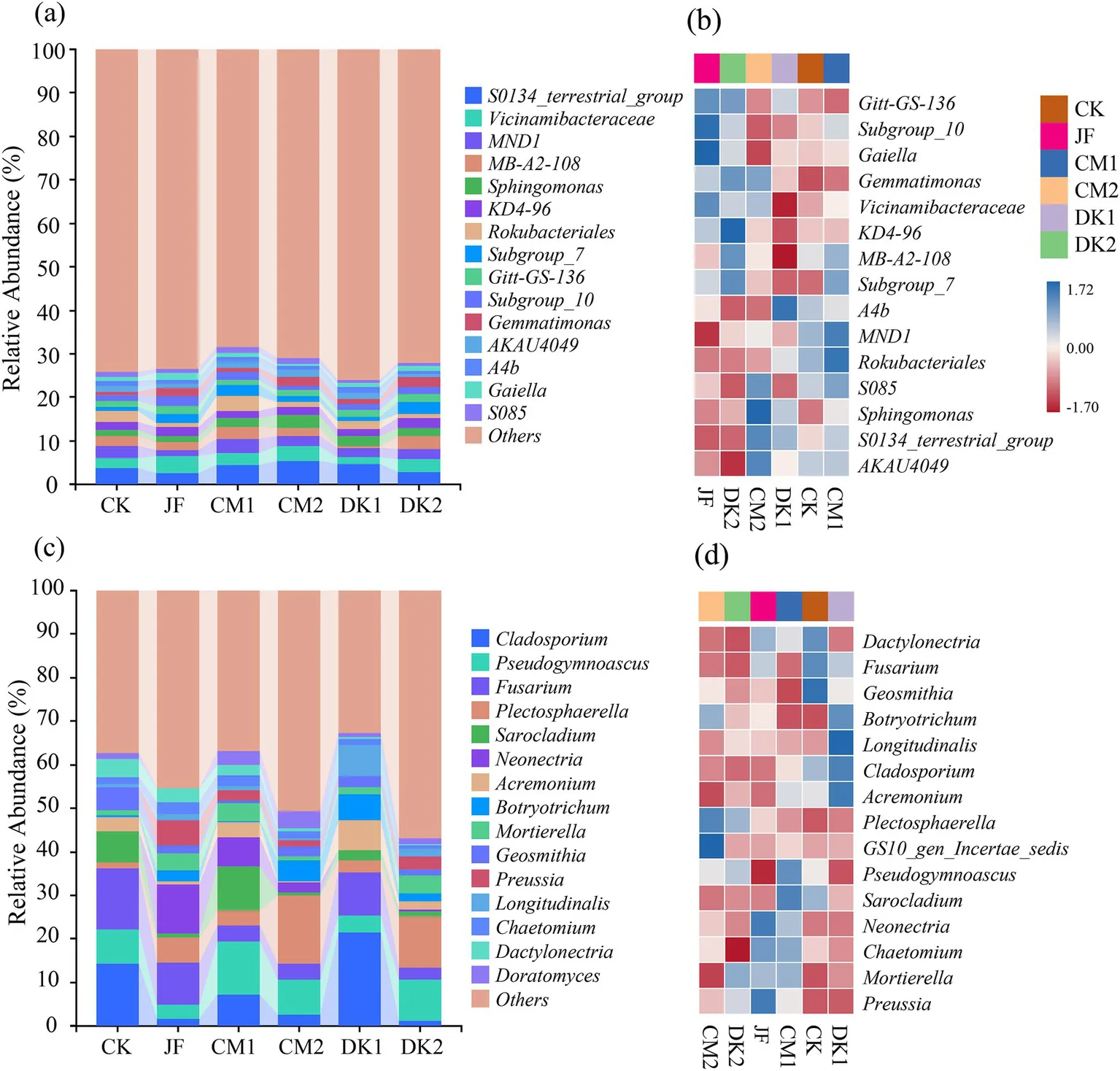
Relative abundance of dominant bacterial taxa **(a)** and fungal genera **(b)** under different fertilization treatments.

At the fungal genus level (Figure 6c), the 15 most abundant genera were *Cladosporium, Pseudogymnoascus, Fusarium, Plectosphaerella, Sarocladium, Neonectria, Acremonium, Botryotrichum, Mortierella, Geosmithia, Preussia longitudinalis, Chaetomium, Dactylonectria,* and *Doratomyces*. CK was dominated by Fusarium (14.03%), whereas JF significantly enriched *Neonectria* (11.22%) and Geosmithia (5.78%). DK1 harbored the highest abundance of *Cladosporium* (21.49%). The potential pathogen *Fusarium* exhibited its maximum abundance in CK (14.03%) and declined markedly across all organic treatments (5.6–9.8%). In terms of density effects, high-density sweet clover (CM2) did not further suppress *Fusarium* but instead elevated the abundances of *Plectosphaerella* and *Doratomyces*. Relative to DK1, DK2 significantly increased the abundances of *Mortierella* and *Pseudogymnoascus*. Collectively, all organic inputs effectively suppressed the soilborne pathogen *Fusarium*, with CM1 demonstrating the greatest efficacy; moreover, high-density oil sunflower planting favored the enrichment of the phosphate-solubilizing fungus *Mortierella*.

### 3.7 Differential analysis of soil bacterial communities

As shown in Figure 7, LEfSe analysis was conducted to characterize the bacterial taxa that responded differently to various fertilization treatments across multiple taxonomic levels. Based on an LDA score >3 and *p* < 0.05, a total of 54 discriminative taxa were identified. The number and composition of differential taxa varied considerably among treatments, reflecting distinct microbial responses to different fertilizer inputs. In the CK treatment, only three differential taxa were detected, including g_Pseudomonas, f_Pseudomonadaceae, and f_Crocinitomicaceae. In contrast, the JF treatment showed the largest number of differential taxa, with 19 taxa identified. Among these, c_Vicinamibacteria, o_Vicinamibacterales, f_Vicinamibacteraceae, c_Thermoleophilia, and g_Vicinamibacteraceae exhibited strong discriminatory contributions, indicating their close association with the JF treatment.

**Figure 7:**
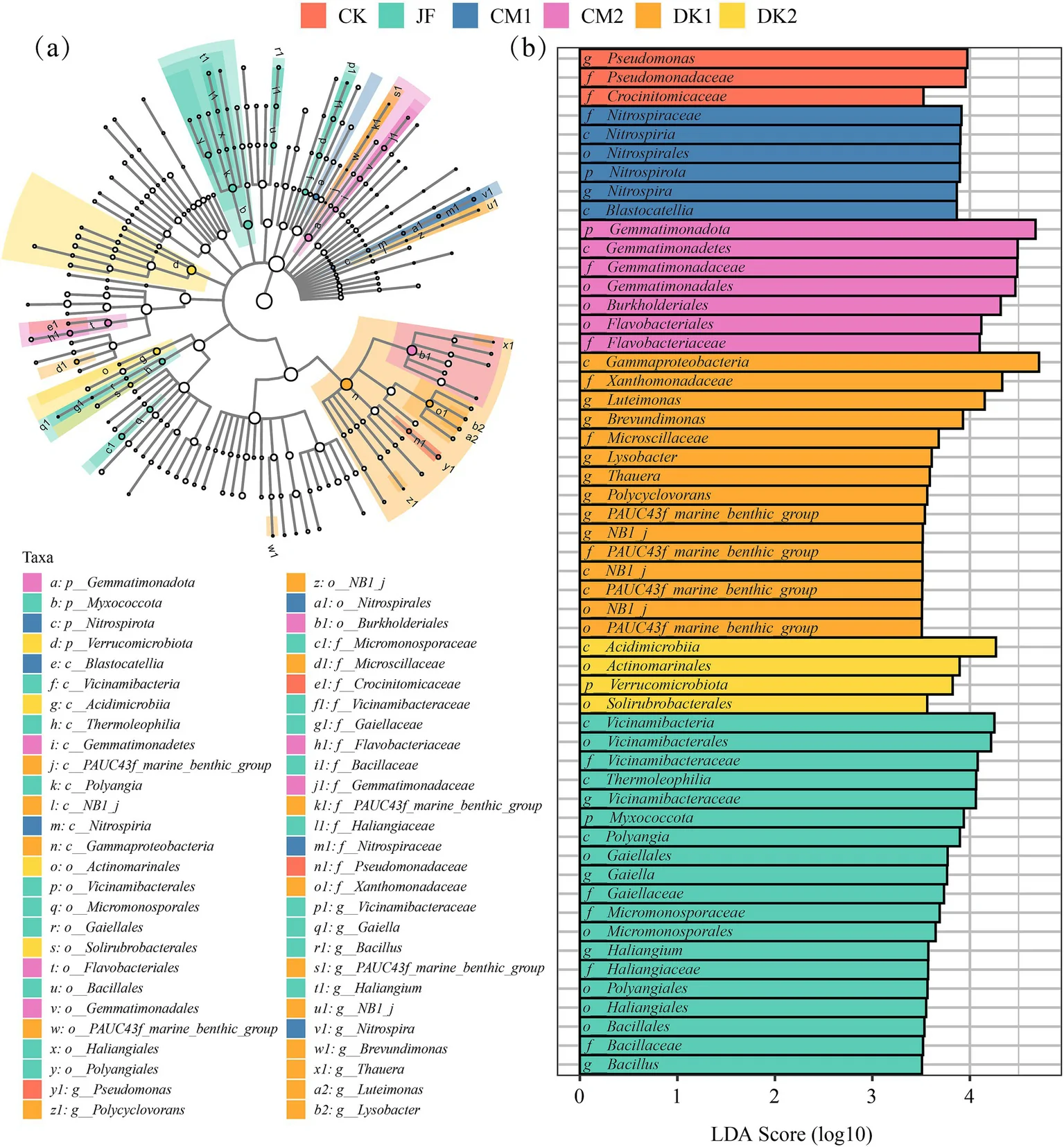
Significantly differential species in soil bacterial communities under different fertilization treatments.

The CM1 treatment showed a distinct enrichment pattern characterized by nitrogen-related bacterial groups. Six differential taxa were identified, including f_Nitrospiraceae, c_Nitrospiria, o_Nitrospirales, and p_Nitrospirota. The CM2 treatment was mainly associated with the enrichment of p_Gemmatimonadota, c_Gemmatimonadetes, f_Gemmatimonadaceae, and o_Gemmatimonadales, with seven differential taxa identified. The DK1 treatment exhibited a broader response, with 15 differential taxa detected. Among them, c_Gammaproteobacteria, f_Xanthomonadaceae, and g_Luteimonas showed relatively high contributions. Compared with other treatments, DK2 exhibited fewer differential taxa, with only four taxa identified, including c_Acidimicrobiia, o_Actinomarinales, p_Verrucomicrobiota, and o_Solirubrobacterales.

The differential responses associated with fertilizer sources suggested distinct ecological preferences of soil bacterial communities. The JF treatment promoted the enrichment of bacterial groups potentially involved in organic matter decomposition, whereas the CM1 treatment favored the accumulation of nitrification-related microorganisms. Meanwhile, DK1 enhanced the abundance of metabolically diverse γ-Proteobacteria-related taxa. Changes induced by fertilizer density were also evident, with CM2 promoting p_Gemmatimonadota-related groups, while DK2 showed an increased representation of Actinobacteria-associated taxa.

To further assess the robustness of differential taxa identified by LEfSe, genus-level bacterial communities were independently analyzed using ANCOM-BC2 (Analysis of Compositions of Microbiomes with Bias Correction 2). This analysis identified 37 significantly differentially abundant genera based on q < 0.05 and diff_robust = TRUE, further demonstrating that fertilization treatments reshaped soil bacterial community composition. By comparing the results from LEfSe and ANCOM-BC2, four genera were consistently detected by both approaches, including g_Nitrospira, g_Pseudomonas, g_Polycyclovorans, and g_PAUC43f_marine_benthic_group. The consistent identification of these genera across two independent analytical methods highlights their stable responses to fertilization treatments and suggests their potential roles as key bacterial indicators associated with fertilizer-induced microbial changes.

The abundance patterns of these four shared responsive genera were further visualized using heatmap and boxplot analyses (Supplementary Figures S5b,c). Among them, g_Nitrospira displayed a clear enrichment pattern under the CM1 treatment, which was consistent with the significant contribution of Nitrospira-related taxa identified by LEfSe analysis. This result suggests that CM1 treatment may favor the establishment of microbial groups associated with nitrogen cycling. In comparison, g_Pseudomonas, g_Polycyclovorans, and g_PAUC43f_marine_benthic_group exhibited treatment-dependent variations in abundance, indicating that different fertilizer inputs may alter microbial niches and resource utilization patterns in soil bacterial communities.

The LEfSe cladogram (Figure 7a) further illustrated the phylogenetic distribution patterns of treatment-associated bacterial taxa. In the CK treatment, characteristic taxa were mainly concentrated within g_Pseudomonas-related groups. The JF treatment displayed the broadest enrichment of discriminative taxa, spanning multiple taxonomic levels from phylum to genus, with coordinated enrichment of Vicinamibacteria-related taxa and Bacillales-associated groups as a prominent feature. The CM1 treatment showed clear enrichment of nitrogen cycling-related microorganisms, among which g_Nitrospira was jointly identified by LEfSe and ANCOM-BC2 analyses, providing further evidence for its stable response to nitrogen inputs. The CM2 treatment exhibited coordinated enrichment of p_Gemmatimonadota and related functional bacterial groups. The DK1 treatment was characterized by extensive enrichment of c_Gammaproteobacteria and associated taxa, whereas the characteristic taxa in DK2 were mainly linked to c_Acidimicrobiia and Actinobacteria-related groups.

### 3.8 Differential analysis of soil fungal communities

As shown in Figure 7, LEfSe analysis was performed on soil fungal communities at both phylum and genus levels, identifying 32 taxa with significant differential abundance (LDA > 3, *p* < 0.05). CK soil revealed only two significantly differential taxa, specifically *f_Leptosphaeriaceae* and *g_Leptosphaeria*. From JF, a total of four differential taxa were identified, comprising *c_Pezizomycetes, o_Pezizales, f_Pyronemataceae,* and *g_Flavocillium*. CM1 soil contained four differential taxa, including *f_Hydnodontaceae, g_Subulicystidium, o_Trechisporales,* and *g_Mytilinidales*_gen_Incertae_sedis. CM2 soil harbored six differential taxa: *p_Rozellomycota, f_GS10_fam_Incertae_sedis, g_GS10_gen_Incertae_sedis, o_GS10, c_Rozellomycotina_cls_Incertae_sedis*, and *g_Bryochiton*. DK1 soil displayed only a single differential taxon, g_Acaulium. DK2 soil exhibited 15 differential taxa, among which *c_Orbiliomycetes, f_Orbiliales_fam_Incertae_sedis, g_Orbiliales_gen_Incertae_sedis, o_Orbiliales,* and *o_Coniochaetales* were the most prominent. Notably, CM2 specifically provoked an outburst of *Rozellomycota*—a hallmark feature of this treatment—whereas DK2 established a multi-functional fungal network encompassing nematode predation, insect parasitism, and organic matter decomposition, in marked contrast to the extreme paucity of biomarkers observed in DK1.

To further examine whether the fungal biomarkers identified by LEfSe were robust, we performed an independent differential abundance analysis using ANCOM-BC2 at the genus level. The results showed that fertilization treatments reshaped the composition of soil fungal communities, with multiple genera exhibiting significant responses to different fertilization regimes. When the results from LEfSe and ANCOM-BC2 were compared, several genera were repeatedly identified by both approaches. Their consistent responses across analytical methods suggest that these taxa may represent relatively stable fungal indicators associated with fertilization-driven shifts in soil fungal community structure.

The heatmap and boxplot based on the shared responsive genera (Supplementary Figure S6b) provided a clearer view of how these taxa responded under different fertilization treatments. Among them, Mortierella displayed distinct variations in relative abundance across fertilization regimes, suggesting a sensitive response to changes in fertilizer inputs. Leptosphaeria and Cladosporium also exhibited treatment-dependent abundance patterns, further supporting their potential as stable responsive fungal taxa. These findings indicate that fertilizer application influenced not only the overall composition of soil fungal communities but also the relative distribution of specific fungal groups, which may contribute to shifts in fungal ecological niches.

The cladogram analysis (Figure 8a) further illustrated the taxonomic distribution of characteristic fungal groups under different fertilization treatments. In the CK treatment, the indicator taxa were mainly associated with the family Leptosphaeriaceae, reflecting the dominant fungal features under unfertilized conditions. The JF treatment was characterized by the enrichment of taxa belonging to the class Pezizomycetes. The CM1 treatment showed a notable enrichment of Basidiomycota-related saprotrophic fungi, whereas the CM2 treatment was mainly characterized by the increased representation of Rozellomycota. Compared with other treatments, DK1 exhibited relatively few indicator taxa, suggesting a less pronounced fungal enrichment pattern. In contrast, DK2 promoted the enrichment of fungal groups with potential ecological roles in predation, parasitism, and organic matter degradation.

**Figure 8:**
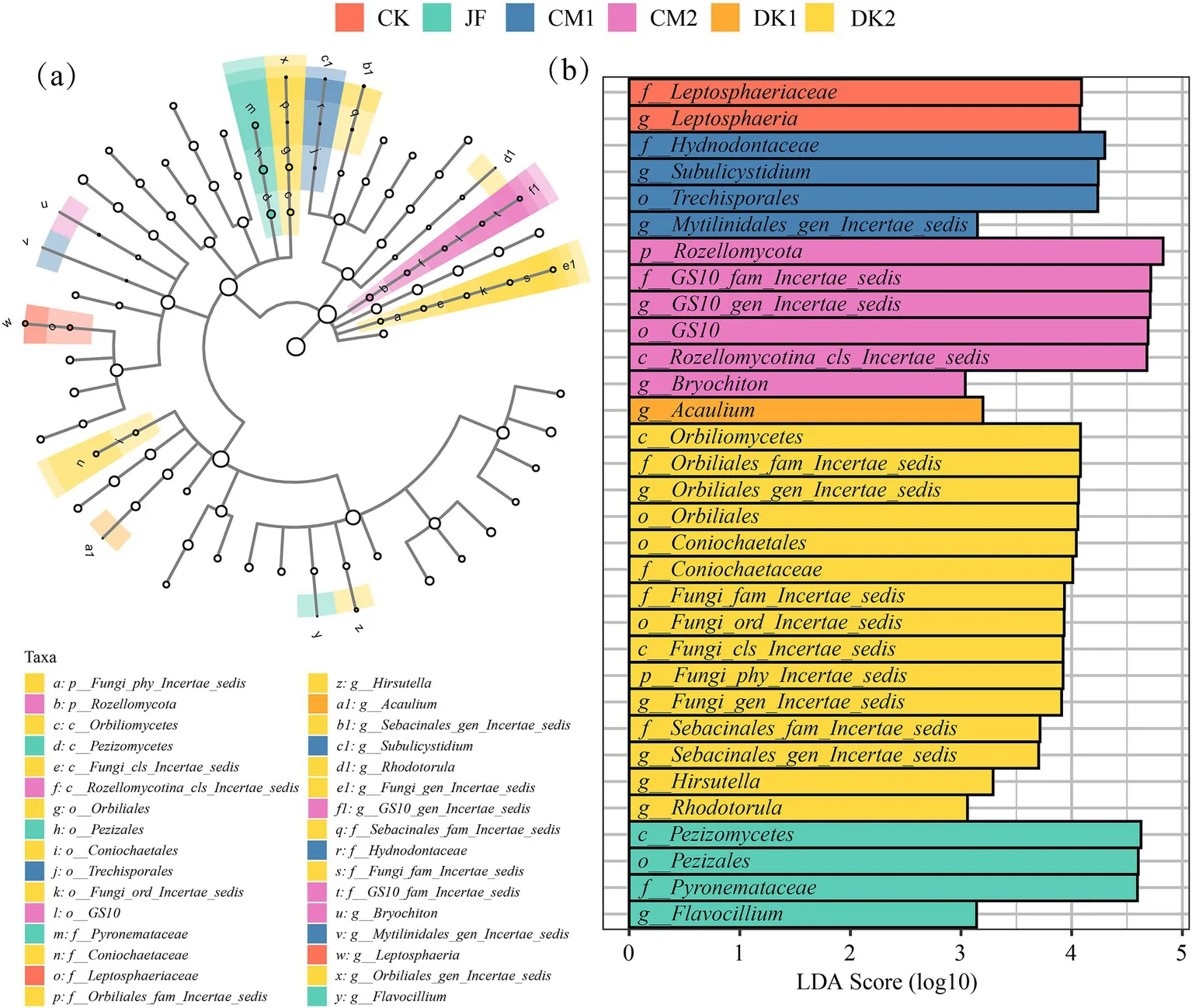
Significantly differential species in soil fungal communities under different fertilization treatments.

### 3.9 Predicted microbial functional pathways and abundance of key functional genes

Changes in predicted bacterial functional pathways and the abundances of their associated key functional genes are shown in Supplementary Figure S2 and Figure 9.

**Figure 9:**
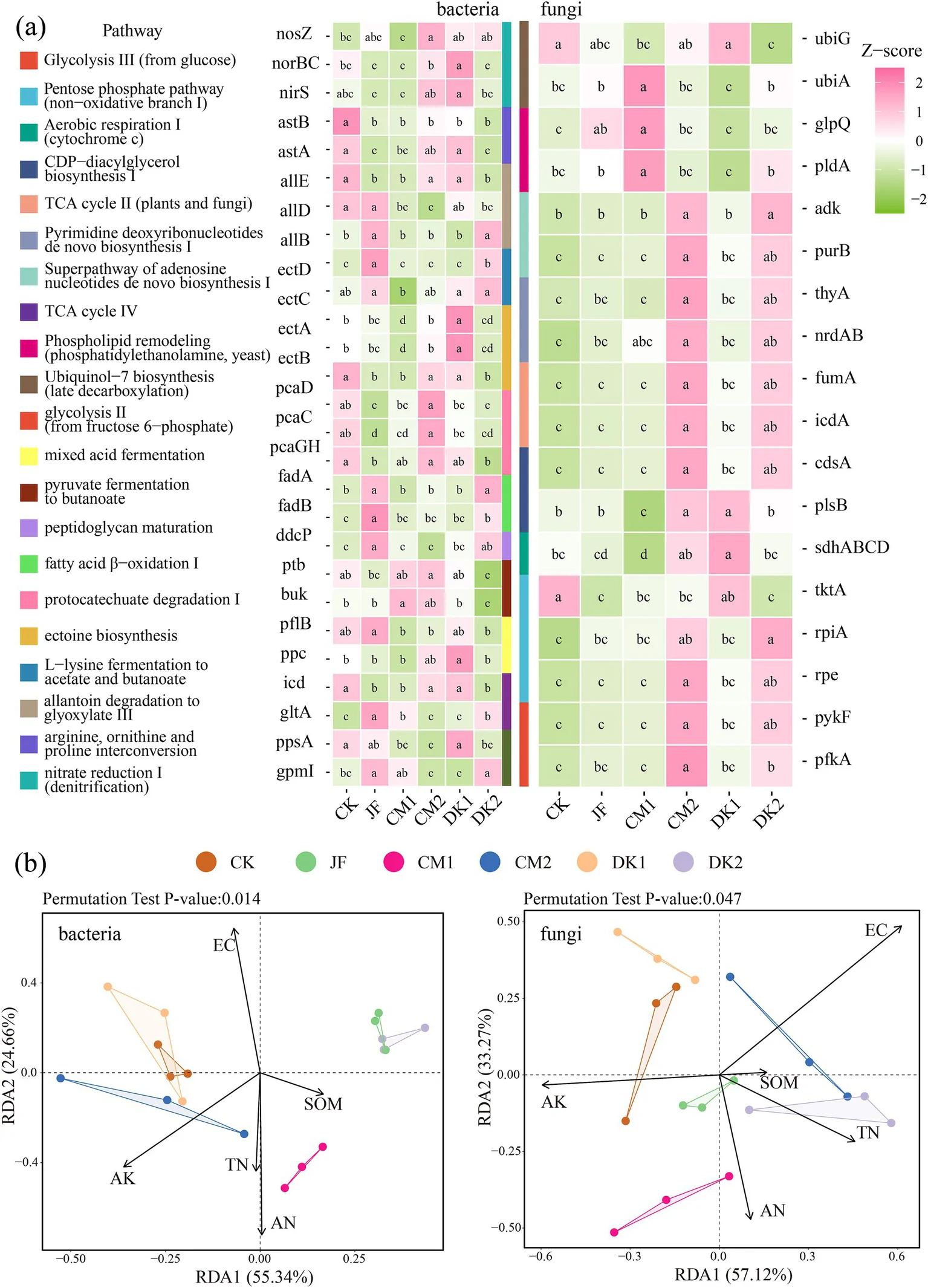
Z-score heatmap of microbial metabolic functional genes **(a)** and redundancy analysis (RDA) of functional gene abundances with soil physicochemical factors under different fertilization treatments **(b)**. EC, Electrical conductivity; SOM, Soil organic matter; AK, Available potassium; AN, Alkaline hydrolysis nitrogen; TN, Total nitrogen.

With respect to carbon metabolism and energy supply, the JF treatment exhibited a higher predicted potential for fatty acid β-oxidation I. The predicted abundance of the associated key gene fadA increased significantly by 11.86 and 12.91% under the JF and DK2 treatments, respectively, compared with CK (*p* < 0.05). In contrast, the predicted abundance of the fatty acid β-oxidation I pathway decreased by 5.19% under CM1 relative to CK (*p* < 0.05). Furthermore, the predicted abundance of the pflB gene associated with mixed acid fermentation increased by 14.21% under JF compared with CK (*p* < 0.05), suggesting that the JF treatment may enhance the predicted functional potential associated with mixed-acid fermentation. The CM1 treatment showed an increasing trend in the predicted functional potential related to butyrate fermentation. The predicted abundance of the pyruvate fermentation to butanoate pathway increased by 19.77% under CM1 compared with CK, accompanied by a 26.76% increase in the associated key gene buk (*p* < 0.05), indicating that CM1 may promote the predicted functional potential associated with butyrate fermentation. Meanwhile, the predicted abundance of the key gene gltA involved in TCA cycle IV increased by 4.95% relative to CK (*p* < 0.05), suggesting an enhancement of the predicted potential functions associated with the TCA cycle under CM1 treatment. Under DK1, the predicted abundances of pathways associated with the TCA cycle IV and glycolysis II increased significantly by 7.49 and 2.50%, respectively, compared with CK (*p* < 0.05).

Predicted abundances of genes involved in nitrogen metabolism exhibited distinct patterns among fertilization treatments.

Compared with CK, the predicted abundance of the L-lysine fermentation to acetate and butanoate pathway increased by 175.42% under JF, while the predicted abundance of its associated key gene ectD increased by 203.32% indicating that JF treatment altered the predicted functional potential associated with amino acid fermentation. Under CM1, the predicted abundances of denitrification-related functional genes were reduced. The predicted abundances of the denitrification-related genes nirS and norBC within the nitrate reduction I pathway decreased by 12.73 and 40.32%, respectively (*p* < 0.05). In addition, the predicted abundance of the allantoin degradation to glyoxylate III pathway, which is associated with organic nitrogen mineralization, also decreased significantly under CM1. Under CM2, the predicted abundance of the terminal denitrification gene nosZ increased significantly by 51.30% compared with CK (*p* < 0.05). In DK1, the predicted abundance of the nitrate reduction I (denitrification) pathway increased by 38.65%, accompanied by coordinated increases in the predicted abundances of nirS and norBC.

The predicted nitrogen functional profile of DK2 was intermediate between those of JF and DK1. Specifically, the predicted abundance of the L-lysine fermentation to acetate and butanoate pathway increased by 111.43%, whereas within the nitrate reduction I (denitrification) pathway only the terminal gene nosZ showed a significant increase (32.15%, *p* < 0.05). Regarding predicted pathways related to stress response and aromatic compound degradation, the predicted abundance of the stress-response pathway decreased by 40.70% under CM1 compared with CK, while the predicted abundances of its two associated key genes decreased by 40.97 and 43.49%, respectively (*p* < 0.05). This pattern coincided with the lowest soil electrical conductivity observed under the CM1 treatment. For aromatic compound degradation, the predicted abundance of the protocatechuate degradation I pathway decreased by 43.07 and 32.34% under the JF and DK2 treatments, respectively, relative to CK. Likewise, the predicted abundances of the three associated key genes decreased significantly by 36.17–52.40% (*p* < 0.05).

Changes in predicted fungal functional pathways and the abundances of their associated key functional genes are presented in Supplementary Figure S3 and Figure 9.

Changes in predicted fungal functional pathways and the abundances of associated key functional genes are shown in Supplementary Figure S3 and Figure 9. Regarding carbon metabolism and aerobic respiration, the CM2 and DK2 treatments significantly increased the predicted functional potentials associated with fungal Glycolysis III and TCA cycle II pathways. Compared with CK, the predicted abundances of the glycolysis III and TCA cycle II pathways increased by 9.70 and 8.49%, respectively, under CM2. Correspondingly, the predicted abundances of the glycolysis-associated genes pfkA and pykF increased significantly by 13.62 and 9.60%, respectively, while those of the TCA cycle II-associated genes icdA and fumA both increased by 9.62%.

A similar pattern was observed under DK2. Relative to CK, the predicted abundances of the glycolysis III and TCA cycle II pathways increased significantly by 7.13 and 7.23%, respectively (*p* < 0.05). Meanwhile, the predicted abundances of pfkA and pykF increased by 7.77 and 7.23%, respectively, whereas those of icdA and fumA increased by 115.86 and 7.23%, respectively.

In addition, the predicted abundance of the aerobic respiration I pathway increased by 8.23% under CM2 and by 10.66% under DK1 relative to CK. Consistently, the predicted abundance of the associated gene cluster sdhABCD increased significantly by 4.49 and 9.27%, respectively (*p* < 0.05). In contrast, the predicted abundance of the aerobic respiration I pathway decreased significantly by 11.76% under CM1 compared with CK (*p* < 0.05).

Regarding membrane lipid remodeling and signal transduction, the CM1 treatment significantly increased the predicted functional potential associated with membrane lipid remodeling. Compared with CK, the predicted abundance of the phospholipid remodeling pathway increased by 113.19% under CM1. Meanwhile, the predicted abundances of its associated key genes pldA and glpQ increased significantly by 120.60 and 33.17%, respectively (*p* < 0.05). Similarly, the predicted abundance of the ubiquinol-7 biosynthesis pathway increased significantly by 149.61% under CM1 (*p* < 0.05), accompanied by a significant increase in the predicted abundance of its associated key gene ubiA.

Predicted pathways involved in nucleotide biosynthesis were enriched under the CM2 treatment. Compared with CK, the predicted abundance of the pyrimidine deoxyribonucleotides *de novo* biosynthesis I pathway increased significantly by 7.50%, while the predicted abundances of its associated key genes nrdAB and thyA increased by 9.79 and 8.45%, respectively.

Likewise, the predicted abundance of the superpathway of adenosine nucleotides *de novo* biosynthesis I increased significantly by 8.76%, accompanied by increases of 9.62 and 8.80% in the predicted abundances of the associated key genes purB and adk, respectively (*p* < 0.05).

Collectively, distinct patterns were observed in the predicted functional profiles of bacterial and fungal communities across the different fertilization treatments.

The predicted bacterial functional profiles varied primarily in pathways associated with carbon metabolism, nitrogen transformation, and stress adaptation. Compared with the other treatments, JF and DK2 exhibited relatively higher predicted abundances of pathways related to fatty acid β-oxidation I and L-lysine fermentation to acetate and butanoate, whereas CM1 was characterized by a higher predicted abundance of the pyruvate fermentation to butanoate pathway together with lower predicted abundances of pathways associated with nitrate reduction I (denitrification) and ectoine biosynthesis. In contrast, DK1 exhibited relatively higher predicted abundances of pathways related to glycolysis II, TCA cycle IV, and nitrate reduction I (denitrification), while CM2 was characterized by an increased predicted abundance of the terminal denitrification gene nosZ.

Overall, different fertilization strategies reshaped the predicted functional profiles of bacterial and fungal communities, leading to distinct microbial metabolic responses. Bacterial functional alterations were primarily linked to pathways involved in carbon metabolism and nitrogen cycling. The JF and DK2 treatments promoted higher predicted potentials for fatty acid β-oxidation and fermentation-related processes, whereas CM1 mainly enhanced functions associated with butyrate fermentation. Meanwhile, variations in green manure planting density further influenced the composition of nitrogen cycling-related functional genes, suggesting that biomass input intensity could modify microbial nutrient transformation potential.

Compared with bacteria, fungal functional responses appeared to be more closely associated with green manure species and planting density. The CM2 and DK2 treatments increased the predicted abundance of functions related to glycolysis, the TCA cycle, and nucleotide metabolism, while CM1 showed enhanced potentials for membrane lipid remodeling and ubiquinone biosynthesis. These results suggest that exogenous microbial fertilizer and different green manure management strategies did not simply increase microbial activity but instead selectively shaped community functional profiles, driving divergent metabolic adaptation patterns within soil microbial communities.

### 3.10 Redundancy analysis of microbial metabolic pathways and environmental factors

For bacterial metabolic pathways, the first two RDA axes (Supplementary Figure S4a) collectively explained 53.84 and 26.78% of the observed variation, respectively, with a cumulative explanatory power of 80.62%. For fungal metabolic pathways (Supplementary Figure S4b), the corresponding axes accounted for 68.99 and 22.20% of the variation, yielding a cumulative explanation rate of 91.19%. Available potassium (AK), alkaline hydrolysis nitrogen (AN), and total nitrogen (TN) emerged as the primary factors shaping bacterial pathway profiles, whereas fungal pathways were predominantly influenced by electrical conductivity (EC), AN, and TN. Bacterial pathway abundances in CK, CM1, CM2, and DK1 treatments tended to be positively associated with AK, AN, and TN; in contrast, those in JF and DK2 exhibited negative correlations with these parameters. The permutation test (*p* = 0.65), however, indicated that fungal metabolic pathways were not significantly affected by soil physicochemical properties.

At the gene level, RDA of bacterial functional genes (Figure 9b) revealed that the first two axes explained 55.34 and 24.66% of the variation, with a cumulative explanatory capacity of 79.99%. For fungal functional genes (Figure 9b), the first two axes accounted for 57.12 and 33.27% of the variation, achieving a cumulative explanation rate of 90.39%. Similar to the pathway-level analysis, AK, AN, and TN were the principal environmental determinants of bacterial functional gene composition, whereas fungal functional genes were primarily regulated by EC, AN, and TN. Bacterial functional genes in CK and DK1 treatments were predominantly governed by AK and EC, while those in CM1 showed positive correlations with AN and TN. Notably, bacterial functional genes in JF, DK2, and CM2 treatments were positively correlated with soil organic matter (SOM) but were not regulated by AK. Fungal functional genes in CK and JF treatments exhibited positive associations with AK. Furthermore, bacterial functional genes in CM1 were more closely aligned with AN, whereas those in CM2 and DK2 were more strongly associated with EC, SOM, and TN.

## 4 Discussion

### 4.1 Effects of exogenous biofertilizer and endogenous green manure on soil microbial diversity in pear orchards

Soil bacteria and fungi play fundamental roles in regulating nutrient cycling, sustaining soil productivity, promoting plant growth, and maintaining ecosystem stability (Van Der Heijden et al., 2008; Yang et al., 2018; Trivedi et al., 2020). Both microbial fertilizers and green manure have therefore been widely adopted as sustainable management practices to improve soil physicochemical properties and enhance soil microbial diversity.

In the present study, microbial fertilizer significantly increased fungal α-diversity, whereas its effect on bacterial diversity was comparatively limited. This observation is consistent with the mechanistic study of Wu et al. (2022), who reported that *Bacillus subtilis* and *Bacillus licheniformis*, the dominant functional microorganisms in microbial fertilizers, secrete extracellular enzymes, including proteases and cellulases, together with B vitamins that facilitate the degradation of complex organic substrates into readily available low-molecular-weight compounds. Such changes may broaden the diversity of carbon and nitrogen resources available to saprotrophic fungi, thereby creating additional nutritional niches and promoting fungal diversity (Wang et al., 2021; Zhong et al., 2021). In contrast, bacterial community composition is generally considered to be more strongly constrained by long-term environmental filters, such as soil pH and salinity, and may therefore be less responsive to short-term application of a single microbial inoculant. Collectively, these findings suggest that the introduction of exogenous beneficial microorganisms may exert a stronger influence on fungal communities than on bacterial communities, potentially owing to the broader substrate utilization capacity and rapid hyphal-mediated responses of fungi.

Although the CM1 treatment maintained intermediate bacterial and fungal α-diversity, its β-diversity differed markedly from that of the other treatments, indicating a pronounced shift in community composition rather than species richness. Duan et al. (2024)reported that leguminous green manure increased both microbial abundance and diversity while altering community composition, whereas Lyu et al. (2025) found that incorporating green manure under conservation tillage reduced bacterial α-diversity but promoted the proliferation of dominant functional taxa, ultimately improving soil quality. The latter finding is broadly consistent with the present results. CM1 created a more favorable soil environment, characterized by higher nutrient availability, the highest alkali-hydrolyzable nitrogen content, and the lowest EC value among all treatments, whereas DK1 resulted in the greatest increase in soil organic matter. These results suggest that improved soil fertility may not always coincide with increased microbial diversity, as variations in nutrient availability and environmental conditions can reshape microbial community composition. Under conditions of enhanced nutrient availability and reduced salt stress, microbial communities may undergo compositional shifts, favoring the enrichment of specific taxa better adapted to the altered soil environment. Such community reassembly may influence the distribution of dominant microbial groups and contribute to changes in nutrient transformation processes (Louca et al., 2018). This interpretation is further supported by the NMDS analysis, in which the bacterial communities of CM1 and CM2 clustered closely together but were clearly separated from those of JF and DK1, suggesting that fertilizer type may represent a more important determinant of bacterial community composition than planting density.

Density-dependent responses differed markedly between the two green manure species. Increasing the planting density of *Melilotus officinalis* significantly reduced bacterial Shannon diversity, whereas increasing sunflower density resulted in its recovery. This contrasting pattern was accompanied by opposite trends in soil electrical conductivity and the predicted abundance of the nitrate reduction I (denitrification) pathway. Specifically, In CM2, the increased EC and elevated predicted abundances of denitrification-related functional indicators occurred together with a reduction in bacterial Shannon diversity, suggesting that these environmental changes may be related to the decline in bacterial diversity. By contrast, DK2 showed lower EC and reduced indicators associated with potential nitrogen loss, which may have provided relatively favorable conditions for maintaining bacterial diversity. These results indicate that the effects of green manure density on bacterial communities may be partly associated with changes in soil microenvironmental conditions, although the specific mechanisms involved require further validation.

Compared with bacterial communities, fungal β-diversity exhibited weaker separation among treatments, with partial overlap observed between CK and DK1. This pattern may reflect the greater buffering capacity of fungal hyphal networks, which are generally considered less sensitive to short-term fluctuations in soil solution chemistry and more strongly influenced by persistent factors such as plant species (de Vries et al., 2018). Nevertheless, fungal communities under JF, CM1, and DK2 occupied distinct positions in the NMDS ordination, indicating that fungal community composition responded more sensitively to green manure species and planting density than did bacterial communities. This interpretation is further supported by the LEfSe analysis, in which DK2 was enriched with the largest number of fungal biomarkers, suggesting that fungal communities may serve as more responsive indicators of green manure management practices.

### 4.2 Effects of exogenous biofertilizer and endogenous green manure on soil microbial community structure

The composition of soil microbial communities plays a fundamental role in regulating soil ecological processes. At the bacterial phylum level, *Proteobacteria* remained the dominant phylum across all treatments. Its relative abundance was highest under the low-density sunflower treatment (DK1) but decreased under microbial fertilizer (JF) and high-density sunflower (DK2). Proteobacteria are generally regarded as copiotrophic microorganisms that contribute substantially to organic matter decomposition and biological nitrogen fixation, with several taxa capable of converting atmospheric nitrogen into plant-available forms, particularly in nitrogen-limited soils, thereby promoting plant growth (Yang et al., 2023; Li J. et al., 2025). Similarly, Wei et al. (2026) demonstrated that long-term green manure cultivation significantly reshaped bacterial community composition in northwestern China.

In the present study, *Proteobacteria* exhibited distinct responses to different fertilization treatments. Its relative abundance remained lower under JF and DK2, whereas DK1 showed the highest enrichment. The elevated abundance of Proteobacteria in low-density sunflower treatment was mainly attributed to the strong accumulation of *Gammaproteobacteria*. Previous work by Tejeda-Agredano et al. (2013) showed that Asteraceae plants could stimulate the biodegradation of polycyclic aromatic hydrocarbons (PAHs) in soil, which subsequently favored the enrichment of Proteobacteria taxa. This finding aligns with the increased Proteobacteria abundance observed in the sunflower treatments in our study, considering that sunflower is also a member of the Asteraceae family. The enrichment of these bacterial groups may be associated with changes in soil organic matter inputs and resource availability induced by sunflower cultivation, which could provide more suitable ecological niches for *Gammaproteobacteria* involved in the transformation of complex organic compounds. These results further suggest that green manure species selection can influence soil microbial assembly by modifying the resource environment, rather than merely through differences in biomass input.

The elevated abundance of Proteobacteria observed under DK1 was primarily attributable to the enrichment of *Gammaproteobacteria*. Tejeda-Agredano et al. (2013) reported that members of the Asteraceae stimulate the biodegradation of polycyclic aromatic hydrocarbons (PAHs), thereby enriching Proteobacteria in the rhizosphere. A comparable pattern was observed in the present study, suggesting that sunflower root exudates may selectively recruit Gammaproteobacteria with the potential to degrade relatively complex organic substrates.

Conversely, the decline in *Proteobacteria* under JF and DK2 coincided with a marked increase in the relative abundance of *Actinobacteriota*. Similar enrichment of *Actinobacteriota* following sunflower cultivation has been reported by Li et al. (2021), whereas Tejeda-Agredano et al. (2013) found no significant changes under other Asteraceae species, indicating that the response may vary with environmental conditions and plant species. *Actinobacteriota* are widely recognized for their capacity to degrade recalcitrant organic compounds, including cellulose and chitin, while simultaneously producing diverse antimicrobial metabolites (Javed et al., 2021). Thus, the increased abundance of Actinobacteria under JF and DK2 may indicate a greater representation of microbial groups involved in complex organic matter transformation, potentially contributing to shifts in soil ecological functions. These findings extend previous studies by suggesting that density regulation of non-leguminous green manure may selectively reshape the relative balance between Proteobacteria and *Actinobacteriota,* thereby influencing soil organic matter transformation processes and disease-suppressive potential.

*Gemmatimonadota*, which are generally considered oligotrophic bacteria, reached their highest abundance under CM2. This enrichment may be associated with the relatively greater input of structural carbon and the more oxygen-limited conditions created by dense incorporation of leguminous biomass. Notably, CM2 also exhibited increased predicted abundance of the terminal gene in the nitrate reduction I pathway, enrichment of the functionally unclassified phylum *Rozellomycota*, and a reduction in *Ascomycota*. Ascomycota play central roles in decomposing lignin and cellulose and contribute substantially to carbon, nitrogen, and phosphorus cycling, thereby enhancing soil fertility (Ma et al., 2013; Ferrari et al., 2021; Jia et al., 2025). The decline of this group may indicate potential changes in microbial taxa associated with organic matter transformation under increased green manure density. These findings suggest that high-density *M. officinalis* reshaped bacterial and fungal community composition as well as their predicted functional profiles, inducing a distinct microbial response pattern rather than simply enhancing the effects observed under low-density cultivation.

At the fungal phylum level, low-density *M. officinalis* produced the greatest increase in *Basidiomycota*, whereas high-density sunflower markedly enriched *Mortierellomycota*. These fungal groups are closely associated with lignin decomposition and organic phosphorus mineralization, respectively. Similar responses were reported by Ding et al. (2021), who observed that leguminous green manure increased the relative abundances of *Ascomycota*, *Basidiomycota*, *Mortierellomycota*, *Chytridiomycota*, and *Glomeromycota* in apple orchards on the Loess Plateau. Such responses may be related to the release of amino acids, sugars, and flavonoids by leguminous roots, which provide favorable substrates for microbial proliferation (Isobe et al., 2001).

At the genus level, microbial fertilizer preferentially enriched *Vicinamibacteraceae* and *Gaiella*, taxa that have been associated with broad-spectrum organic matter decomposition and antimicrobial metabolite production (Albuquerque et al., 2011; Huber and Overmann, 2018). Huang et al. (2023) likewise reported significant enrichment of *Gaiella* following microbial fertilizer application. However, Tan et al. (2024) observed the opposite trend for both taxa, suggesting that their responses may depend on orchard soil characteristics and microbial fertilizer formulations.

In contrast, CM1 did not preferentially enrich typical decomposer taxa but was instead characterized by *MND1*, *Rokubacteriales*, and *Sphingomonas*. Members of *Sphingomonas* possess the capacity to degrade complex aromatic compounds while producing extracellular polysaccharides that promote soil aggregate formation and carbon sequestration (Ye et al., 1996; Costa et al., 2018; Kucuk, 2020). This pattern suggests that low-density *M. officinalis* may influence microbial community composition through changes in rhizosphere carbon inputs and adjustments in microbial resource utilization strategies.

Kaur et al. (2020) reported that high-density leguminous green manure increased microbial abundance together with dehydrogenase, urease, and alkaline phosphatase activities while improving microbial community structure and nutrient availability. However, such responses were not observed under CM2 in the present study, where indicators of potential functional imbalance were detected instead. This discrepancy may reflect differences in soil type, climatic conditions, and initial soil fertility.

Sunflower exhibited a clear density-dependent pattern in bacterial community assembly. Low-density sunflower selectively enriched *A4b*, whereas high-density sunflower enriched several taxa, including *MB-A2-108* and *Subgroup_7*, which have been associated with the degradation of complex organic substrates and carbon pool stabilization. These findings suggest that increasing the planting density of non-leguminous green manure may diversify rhizosphere carbon inputs, thereby promoting the recruitment of functionally complementary decomposer taxa rather than allowing dominance by a single functional group.

Numerous studies have demonstrated that green manure substantially alters fungal community composition (Piotrowska and Wilczewski, 2012; Zhao et al., 2016; Asghar and Kataoka, 2021; Ablimit et al., 2022). In the present study, the effects of planting density on fungal community reassembly were particularly apparent at the genus level. Under low-density sunflower cultivation, the saprotrophic genus *Cladosporium* showed pronounced enrichment. Previous studies have reported that some *Cladosporium* species possess the ability to utilize simple carbon sources (Zalar et al., 2007). Therefore, the increased abundance of *Cladosporium* under low-density sunflower treatment may be related to differences in soil resource conditions resulting from variations in green manure species and planting density.

By contrast, DK2 markedly enriched *Mortierella* and *Pseudogymnoascus*. Mortierella is a well-recognized phosphate-solubilizing fungus capable of mobilizing insoluble phosphorus through the secretion of organic acids and phosphatases (Li et al., 2020), whereas Pseudogymnoascus participates in soil organic matter decomposition and nutrient cycling and has frequently been reported in saline soils (Arenz et al., 2006; Kim et al., 2019). Their simultaneous enrichment under DK2 may indicate that increasing sunflower planting density shifts the microbial functional strategy from preferential utilization of low-molecular-weight carbon substrates toward a more integrated strategy coupling phosphorus solubilization with saprotrophic decomposition of organic matter.

The density response of *M. officinalis* differed from that of sunflower. From CM1 to CM2, the dominant fungal genera shifted from *Pseudogymnoascus* and *Acremonium* to *Plectosphaerella* and *Doratomyces*. Although the ecological functions of the latter genera remain incompletely understood, certain *Plectosphaerella* species have been reported as weak plant pathogens (Han et al., 2020), suggesting that increased planting density may alter fungal community composition in ways that warrant further investigation.

Across all organic fertilization treatments, the relative abundance of the pathogenic genus *Fusarium* declined significantly, supporting previous findings that organic amendments can reduce the risk of soil-borne diseases (Wang et al., 2022; Ahsan et al., 2024). The greatest reduction occurred under CM1. Although microbial fertilizer increased the relative abundance of *Neonectria,* a weakly parasitic fungus, and *Geosmithia*, a saprotrophic genus, the reduction in *Fusarium* relative abundance was less pronounced than that observed under CM1, suggesting that different fertilization strategies may shape fungal community composition through distinct ecological pathways. Likewise, despite the marked enrichment of *Cladosporium* under DK1, Fusarium abundance remained higher than under CM1, indicating that dominance by saprotrophic fungi does not necessarily translate into stronger disease suppression. Taken together, these genus-level results suggest that CM1 provided the most favorable disease-suppressive environment by maintaining the lowest abundance of Fusarium while moderately enriching potentially beneficial fungal taxa, including *Pseudogymnoascus* and *Acremonium*.

### 4.3 Effects of exogenous biofertilizer and endogenous green manure on microbial functional pathways and associated functional genes

Different fertilization treatments led to distinct shifts in microbial carbon metabolism-related predicted functional potentials. Compared with CK, the microbial fertilizer treatment showed a higher predicted potential for carbon oxidation-related functions. The predicted abundance of the fatty acid β-oxidation-related gene *fadA* increased significantly, while the abundances of ppc, associated with mixed-acid fermentation, and *gltA*, a key gene involved in the TCA cycle, also showed increasing trends. These changes suggest that microbial fertilizer application may enhance the predicted capacity of microorganisms to utilize organic carbon substrates, resulting in a carbon metabolism profile characterized by stronger oxidation-related potential (Song et al., 2020; Phares et al., 2022; Liu et al., 2026). RDA analysis showed that the bacterial functional gene composition under JF was negatively correlated with available potassium (AK), suggesting that the variation in carbon metabolism-related functions was not solely driven by soil potassium availability, but may have resulted from the combined effects of soil organic matter conditions and changes in microbial community composition.

Compared with JF, low-density *Melilotus officinalis* exhibited a different carbon metabolism response pattern. The predicted abundance of the fatty acid β-oxidation pathway decreased significantly relative to CK, whereas the predicted abundance of the butyrate fermentation-related pathway and its key gene buk increased. This pattern suggests that low-density *M. officinalis* may have altered the distribution of microbial carbon metabolism-related functional potentials, with part of the predicted metabolic capacity shifting from fatty acid oxidation toward fermentation-associated processes. Previous studies have shown that microbial fermentation products, particularly short-chain fatty acids, participate in soil carbon cycling and may influence microbial interactions and soil ecological functions (Browning et al., 2006). Littrell and Butler (2024) and Swilling et al. (2021) further reported that organic acid metabolites, such as butyrate, could modify soil microenvironmental conditions and potentially suppress certain soil-borne pathogens. Therefore, the increased predicted potential for butyrate fermentation under low-density *M. officinalis* may reflect changes in microbial metabolite-associated functional profiles. However, because this study did not directly measure organic acid concentrations or pathogen responses, the potential ecological significance of these changes requires further verification.

RDA analysis revealed that the bacterial functional gene composition under low-density *M. officinalis* was positively associated with alkali-hydrolyzable nitrogen (AN) and total nitrogen (TN), indicating that microbial functional changes under this treatment were related to variations in soil nitrogen conditions. As a leguminous green manure, *M. officinalis* has a strong nitrogen input capacity, and its growth and incorporation into soil may increase organic matter inputs while modifying the nutrient availability experienced by microorganisms (Cai and Zhao, 2024). These changes may subsequently influence nitrogen metabolism-related microbial functional potentials. This observation agrees with the findings of Duan et al. (2024), who reported that leguminous green manure could improve soil nitrogen conditions. The results suggest that low-density *M. officinalis* may promote coordinated changes in carbon- and nitrogen-related microbial functional potentials, linking microbial metabolic characteristics with soil nutrient variation. However, because the present study relied on amplicon sequencing-based functional prediction, the actual nitrogen transformation processes and their regulatory mechanisms cannot be directly determined. Further validation using quantitative functional gene analysis and process-level measurements is still required.

Low-density sunflower showed a carbon metabolism response pattern different from that of *M. officinalis*. Under DK1, the predicted functional potentials associated with the TCA cycle and glycolysis increased, whereas butyrate fermentation-related functions did not show significant changes. RDA analysis indicated that the bacterial functional gene composition under DK1 was positively associated with available potassium (AK) and electrical conductivity (EC), suggesting that changes in carbon metabolism-related functions may be linked to variations in soil nutrient and salinity conditions. Potassium plays an important role in microbial growth and metabolic regulation (ARAÚJO et al., 2012; Cui and Tcherkez, 2021; Johnson et al., 2022). Meanwhile, under saline conditions, microorganisms may maintain cellular stability through ion balance regulation and osmoprotectant synthesis, processes that may require additional energy input (Zeng et al., 2022; Xing et al., 2024). Therefore, the increased predicted potentials for glycolysis and the TCA cycle under DK1 may represent a microbial response to altered soil environmental conditions, showing a tendency toward energy acquisition-related metabolic strategies.

In contrast, high-density sunflower exhibited increased predicted potential for fatty acid β-oxidation, whereas the TCA cycle did not show a corresponding increase. This difference suggests that DK2 may rely on a carbon utilization pattern distinct from DK1, with a relatively stronger association with lipid-related substrate metabolism. RDA analysis showed that the functional gene composition under DK2 was positively associated with soil organic carbon (SOC) and negatively associated with AK, indicating that its functional variation may be more closely related to soil organic carbon conditions. Meanwhile, bacterial functional composition under CM2 was also associated with SOC, EC, and TN, although its response pattern differed from that of DK2. These differences suggest that combinations of green manure species and planting densities may shape distinct microbial functional adjustment strategies by altering soil resource environments.

Overall, fertilizer sources, green manure species, and planting densities collectively influenced microbial carbon metabolism-related predicted functional potentials, but different treatments showed distinct response patterns. Microbial fertilizer mainly promoted carbon oxidation-related functional potentials, low-density *M. officinalis* showed stronger fermentation-associated characteristics, whereas low-density sunflower exhibited increased energy metabolism-related functional potentials. These differences reflect the diverse ways in which microbial communities respond to changes in soil resource conditions under different organic input strategies.

Microbial nitrogen metabolism-related predicted functional potentials responded differently to various fertilization treatments. The microbial fertilizer treatment showed a relatively pronounced response in nitrogen metabolism-related functions. Compared with CK, the predicted abundance of the L-lysine fermentation pathway increased significantly, accompanied by an increase in the predicted abundance of the key functional gene *ectD*. This shift suggests that microbial fertilizer input may have altered the predicted functional potentials associated with microbial amino acid metabolism and nitrogen cycling. Previous studies have shown that *Bacillus* species possess strong abilities in organic matter degradation and nutrient transformation (Luo et al., 2024), indicating that their introduction may contribute to changes in soil microbial functional composition.

Unlike JF, the CM1 treatment exhibited a different pattern of nitrogen metabolism-related responses. The predicted abundances of nitrate reduction-related functional genes decreased, while some predicted potentials associated with organic nitrogen metabolism also showed a declining trend. RDA analysis revealed that the bacterial functional gene composition under CM1 was positively associated with alkali-hydrolyzable nitrogen (AN) and total nitrogen (TN), suggesting that the observed functional changes may be linked to variations in soil nitrogen availability. As a leguminous green manure, *Melilotus officinalis* can increase organic matter inputs during plant growth and subsequent soil incorporation, thereby modifying the nutrient environment available to microorganisms and influencing nitrogen metabolism-related functional potentials. This pattern agrees with the findings of Duan et al. (2024), who reported that leguminous green manure could improve soil nitrogen status. The results further suggest that low-density *M. officinalis* may influence soil nitrogen cycling through changes in microbial functional composition. However, because the present study relied on amplicon sequencing-based functional prediction, the actual nitrification and denitrification processes, as well as nitrogen retention efficiency, cannot be directly determined. Further studies combining quantitative functional gene measurements and nitrogen transformation process analyses are needed to clarify these mechanisms.

Changes in green manure planting density also affected nitrogen metabolism-related predicted functional potentials under sunflower cultivation. The nitrogen metabolism profile of DK2 was relatively moderate compared with other treatments. The predicted potential of lysine fermentation increased, and the predicted abundance of the *nosZ*-associated functional gene also increased, whereas other nitrogen cycling-related functional modules did not show consistent responses. This pattern suggests that high-density sunflower cultivation may have altered the composition of microbial nitrogen metabolism-related functional potentials rather than causing a uniform shift across all nitrogen cycling processes. RDA analysis showed that the functional gene composition under DK2 was positively associated with SOC, similar to the pattern observed under JF, indicating that its functional variation may be related to multiple soil environmental factors, particularly organic matter conditions.

In comparison, DK1 and CM2 treatments showed increases in the predicted abundances of some denitrification-related functional genes, indicating that different green manure treatments may modify microbial nitrogen cycling-related functional potentials in distinct ways. RDA analysis showed that the denitrification-related functional potential under DK1 was associated with higher EC and AK levels, whereas CM2 was more closely related to higher AN and TN conditions. These results suggest that changes in soil environmental properties may contribute to the variation of microbial nitrogen metabolism-related functional potentials among treatments. However, because this study used amplicon sequencing-based functional prediction without direct measurements of functional gene abundance or nitrogen transformation processes, these findings only represent potential changes in microbial functional profiles and cannot be directly interpreted as changes in actual denitrification intensity or N₂O emission risk.

Fungal predicted functional profiles responded markedly to differences in green manure species and planting density, with distinct functional patterns emerging among the different treatments. The CM2 and DK2 treatments showed increased predicted abundances of glycolysis, the tricarboxylic acid (TCA) cycle, and aerobic respiration-related functional pathways, suggesting that high-density green manure cultivation may influence energy metabolism-related functional potentials within fungal communities. Previous studies have shown that green manure inputs can alter soil organic matter composition and microbial resource availability, thereby affecting fungal metabolic strategies (Muhammad et al., 2021; Thapa et al., 2021). However, because root exudates, plant biomass, and soil carbon source composition were not directly measured in this study, these functional changes mainly represent differences in the predicted metabolic potential of fungal communities, and the specific environmental factors driving these responses remain to be further clarified.

RDA analysis based on PICRUSt2-predicted functional gene abundances showed that variations in fungal functional composition were mainly associated with soil environmental factors, including EC, AN, and TN, whereas RDA based on the abundance of overall metabolic pathways did not show significant relationships. This pattern suggests that environmental changes may affect specific functional units within fungal communities without necessarily causing broad shifts across the entire metabolic pathway spectrum. The CM2 and DK2 treatments were distributed along the direction of increasing AN and TN, consistent with their relatively higher soil nitrogen levels, indicating that soil nutrient conditions may contribute to the formation of fungal predicted functional profiles.

Compared with high-density treatments, CM1 exhibited a different functional configuration pattern. The predicted abundance of aerobic respiration-related pathways decreased, whereas membrane lipid-related functional pathways increased, suggesting that low-density *Melilotus officinalis* may influence fungal resource utilization patterns and generate functional characteristics distinct from those observed under high-density cultivation. RDA results showed that the predicted abundance of functional genes under CM1 was positively associated with AN and TN but negatively associated with EC, indicating that lower salinity conditions and higher nitrogen availability may be related to the observed functional variations. Nevertheless, because the present study inferred fungal functional profiles based on amplicon sequencing data, these predicted changes cannot be directly linked to specific metabolic processes.

The functional responses of fungi under DK1 were mainly associated with variations in EC. Under this treatment, aerobic respiration-related functional pathways showed increased predicted abundance, whereas some glycolysis- and TCA cycle-related functional units did not exhibit corresponding increases. This response pattern suggests that soil salinity conditions may selectively influence certain fungal functional modules. Previous studies have reported that changes in soil salinity can reshape microbial community composition and functional potentials (Pastor et al., 2009; Nivedita et al., 2021). The present findings further indicate that different combinations of green manure species and planting densities may influence fungal functional composition by modifying soil environmental conditions.

Overall, fungal predicted functional potentials showed clear responses to green manure species and planting density. *Melilotus officinalis* and sunflower exhibited different functional adjustment patterns under different planting densities, with high-density treatments showing relatively stronger energy metabolism-related predicted potentials, whereas low-density *M. officinalis* displayed a distinct functional profile. These results indicate that green manure species and planting density jointly shape fungal community functional potentials. However, the specific ecological processes underlying these changes require further investigation through quantitative functional gene analysis, metabolite measurements, and plant–microbe interaction studies.

## 5 Conclusion

This field experiment systematically examined how exogenous microbial fertilizer and different planting densities of *Melilotus officinalis* and sunflower green manure influenced soil physicochemical properties, microbial community structure, and predicted functional potentials in sandy soils of Korla fragrant pear orchards in southern Xinjiang. The results showed that different organic fertilization strategies reshaped soil microbial ecological characteristics in distinct ways. Although all organic amendments improved the soil environment to varying extents, low-density *M. officinalis* (CM1) exhibited a relatively favorable overall response, with lower soil electrical conductivity, higher soil organic matter and nitrogen availability, and more pronounced changes in microbial community composition. Different fertilization treatments did not simply increase microbial activity but selectively altered microbial community structure. Microbial fertilizer mainly promoted bacterial diversity, whereas green manure species and planting density further influenced the composition and distribution of bacterial and fungal communities. Functional prediction analysis revealed distinct potential metabolic responses among microbial fertilizer, *M. officinalis*, and sunflower treatments. The microbial fertilizer treatment showed increased predicted potentials associated with fatty acid metabolism and amino acid fermentation; CM1 enhanced certain carbon metabolism-related predicted functions while reducing the predicted abundances of some denitrification-related genes; and variations in sunflower planting density affected the composition of predicted functional potentials related to carbon metabolism, nitrogen cycling, and phosphorus transformation. These results suggest that green manure type and planting density may regulate microbial functional responses by altering soil resource conditions and shaping microbial community composition. By integrating soil physicochemical characteristics, microbial community features, and predicted functional profiles, this study revealed that low-density *M. officinalis* (CM1) exhibited relatively strong potential for soil improvement and microbial regulation under the tested conditions, while high-density sunflower and microbial fertilizer treatments also showed certain ecological adjustment effects. The findings emphasize that both green manure selection and planting density play important roles in determining microbial ecological responses in sandy orchard soils of southern Xinjiang, providing a scientific basis for optimizing organic fertilization practices and reducing reliance on chemical fertilizers in Korla fragrant pear cultivation. Future research combining metagenomics, metabolomics, and long-term field observations will help further clarify carbon, nitrogen, and phosphorus cycling processes, as well as microbial functional regulation mechanisms within the microbial fertilizer–green manure–soil–crop continuum, thereby providing stronger support for sustainable orchard management in arid and semi-arid regions.

## Data Availability

The raw sequencing data generated in this study have been deposited in the NCBI Sequence Read Archive (SRA) database under the accession numbers PRJNA1311275 and PRJNA1516937. Sample metadata, ASV feature tables, and taxonomic annotation files are provided as supplementary materials. The input files and output results generated from PICRUSt2 functional prediction analysis are also available to support reproducibility of the functional analysis. All other data supporting the findings of this study are available within the article and its Supplementary materials. Further inquiries can be directed to the corresponding authors.

## Footnotes

1
https://unite.ut.ee/

2
http://www.arb-silva.de/

3
https://www.genescloud.cn/
