# Supplementary material for: Effects of exogenous microbial fertilizer and in situ green manure management on soil microbial community structure and predicted functional potential in Korla fragrant pear orchards
Source: Front Microbiol. 2026 Oct 5;17:1941612. doi: 10.3389/fmicb.2026.1941612 (PMC13677886; doi:10.3389/fmicb.2026.1941612)
Supplement: Supplementary file 1 [file Table_1.DOCX]

**Fig. S1** Effects of different fertilization treatments on soil physicochemical properties.

**Fig. S2** Abundance of bacterial metabolic pathways under different fertilization treatments.

**Fig. S3** Abundance of fungal metabolic pathways under different fertilization treatments.

**Fig. S4** Redundancy analysis (RDA) of microbial metabolic pathway abundances with soil physicochemical factors under different fertilization treatments.

**Fig. S5** Joint identification of treatment‑responsive key bacterial genera using different analytical methods. (a) Venn diagram showing differential bacterial genera screened by ANCOM‑BC2 and LEfSe; (b) Heatmap of relative abundances of the four key bacterial genera jointly identified by the two methods across different treatment groups; (c) Box plots illustrating the relative abundances of *Nitrospira, Pseudomonas, Polycyclovorans*, and *PAUC43f_marine_benthic_group* in different treatment groups.

**Fig. S6** Identification and abundance patterns of shared responsive fungal genera under different fertilization treatments. (a) Venn diagram showing the overlap of fungal genera identified by LEfSe and ANCOM-BC2 analyses. (b) Relative abundance patterns of shared responsive fungal genera identified by LEfSe and ANCOM-BC2 under different fertilization treatments.

**Fig. S1** Effects of different fertilization treatments on soil physicochemical properties.

**Note:**Data are presented as mean ± standard error (n = 3), with a significance level of p = 0.05. Different lowercase letters in the same column indicate significant differences (p < 0.05).

**Fig. S2** Abundance of bacterial metabolic pathways under different fertilization treatments.

**Note:**Data are presented as mean ± standard error (n = 3), with a significance level of p = 0.05. Different lowercase letters in the same column indicate significant differences (p < 0.05).

**Fig. S3** Abundance of fungal metabolic pathways under different fertilization treatments.

**Note:**Data are presented as mean ± standard error (n = 3), with a significance level of p = 0.05. Different lowercase letters in the same column indicate significant differences (p < 0.05).

**Fig. S4** Redundancy analysis (RDA) of microbial metabolic pathway abundances with soil physicochemical factors under different fertilization treatments.

**Fig. S5** Joint identification of treatment‑responsive key bacterial genera using different analytical methods. (a) Venn diagram showing differential bacterial genera screened by ANCOM‑BC2 and LEfSe; (b) Heatmap of relative abundances of the four key bacterial genera jointly identified by the two methods across different treatment groups; (c) Box plots illustrating the relative abundances of *Nitrospira, Pseudomonas, Polycyclovorans*, and *PAUC43f_marine_benthic_group* in different treatment groups.

**Fig. S6** Identification and abundance patterns of shared responsive fungal genera under different fertilization treatments. (a) Venn diagram showing the overlap of fungal genera identified by LEfSe and ANCOM-BC2 analyses. (b) Relative abundance patterns of shared responsive fungal genera identified by LEfSe and ANCOM-BC2 under different fertilization treatments.
