# Supplementary material for: Effects of exogenous microbial fertilizer and in situ green manure management on soil microbial community structure and predicted functional potential in Korla fragrant pear orchards
Source: Front Microbiol. 2026 Oct 5;17:1941612. doi: 10.3389/fmicb.2026.1941612 (PMC13677886; doi:10.3389/fmicb.2026.1941612)
Supplement: Supplementary file 2 [file Table_2.DOCX]

**Note:**CK: Conventional chemical fertilizer (control)，JF: Microbial organic fertilizer treatment, DK1: Low-density sunflower (Helianthus annuus), DK2: High-density sunflower (Helianthus annuus), CM1: Low-density Melilotus officinalis, CM2: High-density Melilotus officinalis, EC :Electrical Conductivity, SOM :Soil Organic Matter, AK :Available Potassium, AN :Alkaline Hydrolysis Nitrogen, and TN :Total Nitrogen
